# In PLN-R14del mice, SR structure restoration, rather than calcium cycling, is the dominant effector of PLN-ASO treatment

**DOI:** 10.1093/cvr/cvaf156

**Published:** 2025-09-04

**Authors:** Liu Sun, Tim R Eijgenraam, Carl Amilon, David Janzén, Kenny M Hansson, Dieter A Kubli, Daniela Später, Adam E Mullick, Peter van der Meer, Vivian Oliveira Nunes Teixeira, Herman H W Silljé

**Affiliations:** Department of Cardiology, University Medical Center Groningen, University of Groningen, Hanzeplein 1, Groningen 9713 GZ, The Netherlands; Department of Cardiology, University Medical Center Groningen, University of Groningen, Hanzeplein 1, Groningen 9713 GZ, The Netherlands; Drug Metabolism and Pharmacokinetics, Research and Early Development, Cardiovascular, Renal and Metabolism, BioPharmaceuticals R&D, AstraZeneca, Gothenburg, Sweden; Drug Metabolism and Pharmacokinetics, Research and Early Development, Cardiovascular, Renal and Metabolism, BioPharmaceuticals R&D, AstraZeneca, Gothenburg, Sweden; Bioscience Cardiovascular, Research and Early Development, Cardiovascular, Renal and Metabolism (CVRM), BioPharmaceuticals R&D, AstraZeneca, Gothenburg, Sweden; Ionis Pharmaceuticals, Carlsbad, CA, USA; Bioscience Cardiovascular, Research and Early Development, Cardiovascular, Renal and Metabolism (CVRM), BioPharmaceuticals R&D, AstraZeneca, Gothenburg, Sweden; Ionis Pharmaceuticals, Carlsbad, CA, USA; Department of Cardiology, University Medical Center Groningen, University of Groningen, Hanzeplein 1, Groningen 9713 GZ, The Netherlands; Department of Cardiology, University Medical Center Groningen, University of Groningen, Hanzeplein 1, Groningen 9713 GZ, The Netherlands; Department of Cardiology, University Medical Center Groningen, University of Groningen, Hanzeplein 1, Groningen 9713 GZ, The Netherlands

**Keywords:** Cardiomyopathy, Heart failure, Cardiac remodelling, Genetheraphy

## Abstract

**Aims:**

Phospholamban (PLN) acts as an inhibitory regulator of calcium uptake in the sarco-/endoplasmic reticulum (SR) of cardiomyocytes. The pathogenic variant, PLN-R14del, leads to dilated and/or arrhythmogenic cardiomyopathy. Previous studies demonstrated that PLN-targeting antisense oligonucleotides (ASOs) can mitigate disease progression in mice. However, it remains unclear whether the protective effects of PLN-ASO therapy are due to improved calcium homeostasis or via reduction of abnormal PLN-SR clusters, a hallmark of this disease.

**Methods and results:**

Homozygous PLN-R14del (R14^Δ/Δ^) mice were randomized to injections with various doses of PLN-ASO (3, 7, 15, or 25 mg/kg) or a scrambled control. Consistent with previous findings, R14^Δ/Δ^ mice exhibited severe cardiac dysfunction, myocardial fibrosis, and aberrant SR clusters by 7 weeks of age. ASO-treated R14^Δ/Δ^ mice displayed a dose-dependent preservation of cardiac function with diminished remodelling and extended lifespan. Correspondingly, abnormal PLN-SR clustering was diminished by ASO therapy in a dose-dependent manner, and SR structure returned to a normal state. Calcium dynamics were investigated separately on isolated cardiomyocytes from treated mice. In wild-type (WT) mice, ASO (25 mg/kg) mediated PLN depletion significantly enhanced calcium and contractile dynamics, confirming effective target engagement. In R14^Δ/Δ^ cardiomyocytes, however, ASO treatment showed limited effects on calcium dynamics. Calcium transient decay and sarcomeric shortening were already enhanced in R14^Δ/Δ^ cardiomyocytes compared to WT, suggesting a partial loss of the PLN-R14del calcium inhibitory function. This pre-existing acceleration of calcium dynamics likely accounts for the limited impact of ASO therapy on calcium regulation in R14^Δ/Δ^ mice.

**Conclusions:**

PLN-ASO treatment demonstrated a dose-dependent restoration of SR organization and a concomitant increase in lifespan in PLN-R14del mice. The enhanced SR calcium uptake in PLN-R14del mice suggests a partial loss of inhibitory function, limiting ASO therapy's effects on calcium dynamics. This implies that PLN-ASO therapy acts predominantly via restoration of SR structure in PLN-R14del cardiomyopathy.

## Graphical Abstract

**Graphical Abstract:**
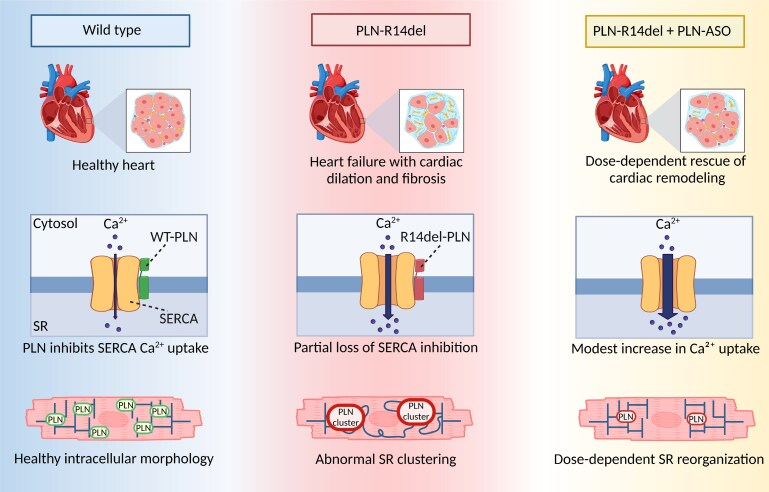

**Time of primary review: 39 days**

**See the editorial comment for this article ‘PLN-R14-del, a puzzling mutation’, by A. Zaza and C. Maniezzi, https://doi.org/10.1093/cvr/cvaf189.**

## 1. Introduction

Phospholamban (PLN) is a small protein that resides in the membrane of the sarcoplasmic reticulum (SR) and is predominantly expressed in ventricular cardiomyocytes. Its primary role is to inhibit the uptake of calcium ions (Ca^2+^) from the cytosol into the SR by interacting with the sarco-/endoplasmic reticulum Ca^2+^-ATPase 2a (SERCA2a) pump.^1,2^ Several pathogenic mutations in the gene encoding PLN have been linked to cardiomyopathies.^2^ Among these, the p.Arg14del (R14del) variant is the most well-known and frequently occurring mutation, which can lead to dilated and/or arrhythmogenic cardiomyopathy, often culminating in severe heart failure (HF).^3^ Currently, there is no specific treatment for this form of cardiomyopathy, and heart transplantation is often required as a last option.^4–6^

Using a PLN-R14del mouse model that has similar disease characteristics as in humans, we recently showed that silencing therapy with an antisense oligonucleotide (ASO), targeting *Pln* mRNA for degradation, could halt disease development and prolong lifespan of PLN-R14del mice.^7^ This was true for a prophylactic treatment, starting before disease development, but also for a therapeutic treatment, starting at the time of early or advanced disease.^7,8^ Therapeutic treatment at the early disease stage could halt further decline in cardiac function, attenuated cardiac fibrosis and hence extended survival of PLN-R14del mice. Moreover, PLN immunohistochemistry showed that dense PLN staining of abnormal SR clusters, a hallmark of this disease,^9^ was observed at the start of treatment but was strongly reduced after ASO treatment.^8^ This might indicate that normal SR structure is restored by ASO treatment, but this needs confirmation beyond PLN (mis)localization. Also, until now, calcium cycling has not been investigated in the context of ASO treatment, while this is particularly interesting for PLN-related cardiomyopathies. Originally, it was thought that PLN-R14del would be a super-inhibitor of SERCA2a.^10^ Badone *et al*.,^11^ however, contradicted this superinhibition theory using human PLN-R14del iPSC derived cardiomyocytes and reported reduced inhibitory activity. Subsequently, also studies in mice carrying a genomic PLN-R14del locus reported reduced inhibitory activity on SERCA2a in cardiomyocytes and this was true for both heterozygous and homozygous PLN-R14del mice.^9,12^ Studies utilizing PLN-targeting antisense therapy could further unravel the impact of PLN silencing on cardiomyocyte calcium handling and SR clustering in PLN-R14del cardiomyopathy, potentially offering deeper insights into the disease mechanism.^13^

Previous studies showed therapeutic effects in PLN-R14del mice using PLN-ASO.^4,7,8^ In this study, we aim to demonstrate the dose-dependent therapeutic effects of a novel PLN-ASO in PLN-R14del mice, with the potential for these findings to translate into meaningful benefits for human PLN-R14del cardiomyopathy. Subsequently, we show for the first time that abnormal SR clustering is resolved and SR organization is rescued in these mice in a dose-dependent manner, which directly correlates with disease progression. Finally, this study shows that PLN-ASO treatment does enhance SR calcium uptake in wild-type (WT) mice, but to a far lesser extent in PLN-R14del mice. Together, these data indicate that the therapeutic effect of PLN silencing in PLN-R14del cardiomyopathy is primarily mediated by improvements in SR structure rather than by enhanced calcium cycling.

## 2. Methods

### 2.1 Study design and animal model

The generation and phenotypic characterization of the PLN-R14del mouse model, and housing of mice have been described before.^5^ Male and female WT and homozygous PLN-R14del (R14^Δ/Δ^) mice were enrolled in this study. Before treatment initiation at 5 weeks of age, baseline echocardiography was performed. Based on sex, weight, and baseline cardiac function, R14^Δ/Δ^ mice were subsequently randomized to six different groups receiving PLN-ASO (5′-GCATATCAATTTCCTG-3′) injections, at a dose of 0 (vehicle only; Dulbecco's PBS), 3, 7, 15, or 25 mg/kg/injection or a scrambled ASO (5′-GGCCAATACGCCGTCA-3′) of 25 mg/kg/injection (*n* = 8 per group). Doses were selected based on dose–response-modelling from previous *in vivo* studies^7,8^ and were chosen to cover PLN inhibition between 20% and 70%. The time to initiate treatment with ASO was informed by previous studies to give optimal opportunity to show a treatment effect. ASOs were designed and produced by Ionis Pharmaceuticals (CA, USA). Six WT mice were included as healthy controls, receiving vehicle injections. Injections started at 5 weeks of age, with four injections in the first week, followed by a weekly maintenance injection of the same dose in the subsequent weeks. R14^Δ/Δ^ mice were monitored until they reached the humane endpoint (ejection fraction (EF) < 10%, >20% weight loss or lethargy and dyspnoea), which generally occurs at 7–8 weeks of age.^5^ In addition, when the average EF of an ASO dosing group was <20% or when >30% of the mice of an ASO dosing group reached the humane endpoint, the entire experimental group was terminated. Surviving R14^Δ/Δ^ groups, together with the WT mice, were kept until 21 weeks of age, with follow-up echocardiograms at 7, 15, and 21 weeks of age. At sacrifice, hearts were collected for histological and molecular analyses. Echocardiography and termination were conducted under anaesthesia with 2.5% isoflurane mixed with oxygen. In a separate experiment for calcium handling and sarcomere measurements in isolated cardiomyocytes, an additional 10 WT and 10 R14^Δ/Δ^ mice were treated with vehicle or 25 mg/kg PLN-ASO between 3 and 5 weeks of age, following the abovementioned regimen. This study was approved by the Dutch Central Authority for Scientific Procedures on Animals (license numbers AVD1050020199105 and AVD10500202115446) and the Animal Welfare Body of the University of Groningen (permit numbers IVD199105-01-011 and IVD2115446-02-003), followed the guidelines set out in the Directive 2010/63/EU of the European Parliament for using animals in research and were reported in line with the Animal Research: Reporting of *In Vivo* Experiments (ARRIVE) guidelines.^14^

### 2.2 Echocardiography

Left ventricular (LV) morphology and function were monitored using a Vevo 3100 preclinical imaging system equipped with a 40 MHz MX550D linear array transducer (FUJIFILM VisualSonics, Canada), as previously described.^9^ Recordings were analysed using Vevo LAB software version 5.7.1 (FUJIFILM VisualSonics). Data acquisition and analysis adhered to the guidelines provided by the European Society of Cardiology Working Group on Myocardial Function.^15^

### 2.3 Euthanasia

Euthanasia was performed under anaesthesia using a mixture of oxygen and 2.5% isoflurane. After ensuring deep anaesthesia, the abdominal and thoracic cavities were opened, blood was collected via the abdominal aorta, and saline was used to flush the circulatory system via the heart. The heart was carefully excised, rinsed in an ice-cold 1 M potassium chloride (KCl) solution (Merck Millipore, Germany). A transverse mid-section of the heart was preserved for histological examination, and the remaining tissue samples were promptly snap-frozen in liquid nitrogen for further molecular analyses.

### 2.4 Histological staining

Transverse cardiac slices of both ventricles were preserved in a 4% formaldehyde solution (10% formalin; Klinipath, The Netherlands) for at least 24 h. After fixation, heart tissues were dehydrated using an automated system (Leica TP1020; Leica Microsystems, Germany), embedded in paraffin, and sectioned into 4 µm thick slices for histological analysis. Tissue sections were deparaffinized before proceeding with staining procedures.

Masson's trichrome staining was performed to identify collagen deposition, adhering to standard protocols. Subsequently, the entire stained sections were scanned using a Nanozoomer 2.0-HT digital slide scanner (Hamamatsu Photonics, Japan). Quantification of fibrosis in each section was accomplished by analysing the percentage of the total section area occupied by fibrotic tissue using the positive Pixel Count v9 algorithm of Aperio's ImageScope software version 12.4.0 (Leica Microsystems).

To evaluate PLN clustering, ventricular tissue sections were stained with a rabbit anti-PLN primary antibody (Cell Signaling Technologies, MA, USA), followed by labelling with an Alexa 555-conjugated secondary antibody. Cell boundaries were visualized using fluorescein isothiocyanate (FITC)-conjugated wheat germ agglutinin (WGA), and nuclei were stained with 4′,6-diamidino-2-phenylindole (DAPI). PLN clusters were quantified in 30–50 cardiomyocytes per mouse (*n* = 6 per group). The cluster-positive cells were presented as per 0.1 mm^2^, as indicated by the scale bar.

To show the colocalization of PLN and other SR proteins in mouse cardiac tissue samples, SERCA2a or HRC (histidine-rich calcium-binding protein) were co-stained with PLN using fluorescently-labelled secondary antibodies. Specific antibodies used in immunohistochemistry are listed in Supplementary material online, *Table S1*.

Fluorescent staining images were acquired using a Leica TCS SP8X confocal microscope (Leica Microsystems). PLN/WGA/DAPI localization and colocalization of SERCA2a or HRC with PLN were captured using a 63× objective (numerical aperture 1.4, pinhole size 95.5 µm).

### 2.5 Quantitative polymerase chain reaction (qPCR)

Total RNA was extracted from snap-frozen ventricular tissue using TRI Reagent (Sigma-Aldrich, MO, USA) according to the manufacturer's protocol. Complementary DNA (cDNA) synthesis was performed using the QuantiTect reverse transcription kit (Qiagen, Germany). Quantitative polymerase chain reaction (qPCR) was conducted with iQ SYBR Green Supermix (Bio-Rad, CA, USA) on a C1000 Thermal Cycler CFX 384 Real-Time PCR Detection System (Bio-Rad). Gene expression levels were analysed using Bio-Rad CFX Manager 3.0 software. All gene expression levels were normalized to the reference gene *Rplp0* (36B4) and reported as fold changes relative to the WT control group. The primer sequences used for qPCR are listed in Supplementary material online, *Table S2*. The qPCR primers used for PLN mRNA quantification were designed downstream of the R14del mutation site. Primer binding and amplification efficiency are therefore similar for the WT and PLN-R14del transcript.

### 2.6 Western blot

Total protein from snap-frozen ventricular tissue was extracted using Radioimmunoprecipitation Assay (RIPA) buffer as described previously.^5^ Protein concentrations were measured using a Pierce bicinchoninic acid (BCA) protein assay kit (Thermo Scientific, MA, USA). Samples were heated to 95°C for 5 min for PLN or 50°C for 15 min for SERCA2a, followed by loading onto a sodium dodecyl sulfate-polyacrylamide gel electrophoresis (SDS-PAGE) system. Equal amounts of protein were loaded, with 5 µg per well for PLN and 10 µg per well for SERCA2a. Proteins separated by SDS-PAGE were then transferred to an immunoblot polyvinylidene fluoride (PVDF) membrane (Bio-Rad) using a semi-dry transfer system (Amersham Biosciences, UK). The total protein level in each sample was determined using the Revert 700 Total Protein Stain Kit (LI-COR Biosciences, NE, USA), which was subsequently used for protein normalization. Membranes were blocked with blocking buffer and incubated overnight at 4°C with a primary antibody, followed by a one-hour incubation at room temperature with a secondary antibody. Detection was carried out using enhanced chemiluminescence with the Western Lightning Ultra Chemiluminescent Substrate (PerkinElmer, MA, USA) and visualized with the Amersham ImageQuant 800 Western blot imaging system (Cytiva, MA, USA). Quantification of target proteins was performed using Image Studio Lite Software version 5.2 (LI-COR Biosciences). Protein expression values were reported as fold changes relative to the WT control group. Primary and secondary antibodies that were used are presented in Supplementary material online, *Table S3*.

Total protein from snap-frozen isolated cardiomyocytes was extracted using RIPA buffer. To quantify PLN protein levels, a 12–230 kDa Separation Module (ProteinSimple, CA, USA) was used on an automated western blot system (Jess Simple Western; ProteinSimple) following the manufacturer's instructions. Protein lysates were used at 1 mg/mL together with an anti-PLN antibody (1:50, Cell Signaling Technologies) and an Anti-Rabbit Detection Module (ProteinSimple). Protein expression was normalized by protein normalization module (ProteinSimple) following the manufacturer's protocol.

### 2.7 Electron microscopy

Electron microscopy (EM) was performed as reported previously.^9,16^ Briefly, LV myocardial tissue was cut into 1 mm^3^ pieces and fixed in EM fixative (2% paraformaldehyde and 2% glutaraldehyde in 0.1 M sodium cacodylate (PH 7.4)) overnight at 4°C. The fixative was subsequently replaced with fresh buffer (0.5% paraformaldehyde and 2% glutaraldehyde in 0.1 M sodium cacodylate, pH 7.4) to allow for prolonged storage and improved preservation of cellular ultrastructure. Post-fixation was carried out using 1% osmium tetroxide and 1.5% potassium ferrocyanide. The samples were then sectioned, mounted on single-slot cupper grids, and contrasted with 4% neodymium acetate. Sections were imaged using scanning transmission electron microscopy (STEM) (Zeiss Supra55, Germany).

### 2.8 Contraction and calcium measurements in isolated cardiomyocytes

Cardiomyocytes were isolated using a Langendorff-free method.^17^ Cardiomyocytes were loaded with the calcium fluorophore Fura-2, AM and single cell calcium transients and sarcomere shortening were recorded using a MultiCell High Throughput system (IonOptix, MA, USA) under pacing conditions (1.5 Hz, 20 V) at 37°C. At least 40 cells were included per mouse (*n* = 5 per group). Measurements were done at baseline and following beta-adrenergic stimulation using 100 nM isoproterenol. Calcium and contractility data were analysed using CytoSolver with default settings, while excluding cells with <5 transients and outliers outside mean ± 1.5 ∗ interquartile range (IQR).

### 2.9 Statistical analyses

Data are presented as means ± standard error of the mean (S.E.M.). Sarcomere length and ratiometric calcium transients are shown as line graphs. Quantification of SR-EM parameters and contractile and calcium parameters are displayed in SuperPlots presenting both individual cell values (transparent dots) and mean values per mouse (solid dots) with the matching colours.^18^ Data of isolated cardiomyocytes were log-transformed and a hierarchical test with Bonferroni correction was applied in R version 4.4.0.^18^ All other statistical analyses were performed using GraphPad Prism version 9.1.0. For statistical analysis of survival curves, a log-rank test was performed. For all other comparisons, nonparametric tests were performed due to small group sizes (*n* = 5–8). Echocardiographic parameters at each timepoint were compared separately to the WT group of the same age using a Kruskal–Wallis test with *post hoc* Dunn's multiple comparisons test. At the 7-week time point, comparisons were made among all PLN-R14^Δ/Δ^ groups. For other assays, a Mann–Whitney test was used to compare vehicle-treated R14^Δ/Δ^ mice to WT mice (to evaluate differences between genotypes), followed by a Kruskal–Wallis test with *post hoc* Dunn's multiple comparisons test for comparisons among the vehicle-treated and ASO-treated R14^Δ/Δ^. *P*-values of <0.05 were considered statistically significant. All data acquisition and analyses were performed in a blinded manner.

## 3. Results

### 3.1 PLN-ASO treatment rescues R14^Δ/δ^ mice from HF and premature death in a dose-dependent manner

**Figure 1:**
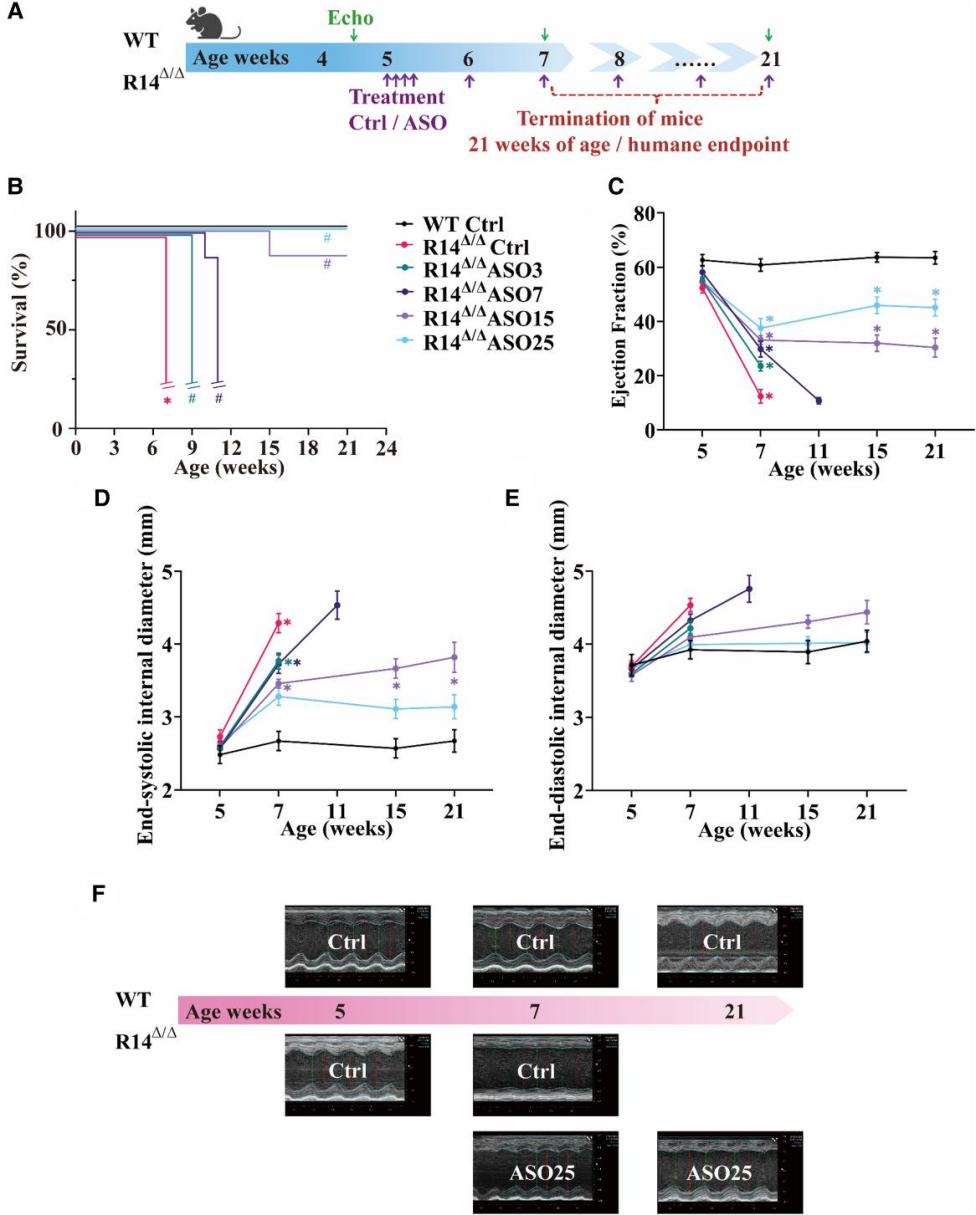
PLN-ASO therapy extends the lifespan and improves cardiac function of PLN-R14^Δ/Δ^ mice. (*A*) Schematic overview of the *in vivo* study design. (*B*) Survival curve of WT mice receiving vehicle injections (Ctrl; *n* = 6) and PLN-R14^Δ/Δ^ mice receiving vehicle injections (Ctrl) or 3, 7, 15, or 25 mg/kg of PLN-ASO (*n* = 8 per group). The interrupted lines of the vehicle and low dose groups indicate that the predefined humane endpoint was reached in these groups. *P = 0.0001 compared to WT Ctrl; ^#^*P* = 0.0001 compared to R14^Δ/Δ^ Ctrl (log-rank test). (*C*) Ejection fraction, (*D*) end-systolic internal diameter, and (*E*) end-diastolic internal diameter of WT mice receiving vehicle injections (Ctrl; *n* = 6) and PLN-R14^Δ/Δ^ mice receiving vehicle injections (Ctrl) or 3, 7, 15, or 25 mg/kg of PLN-ASO (*n* = 8 per group) at 5, 7, 11, 15, and 21 weeks of age. *P < 0.05 compared to WT mice of corresponding age. Kruskal–Wallis with Dunn's multiple comparisons test). (*F*) Representative echocardiographic images of WT Ctrl, PLN-R14^Δ/Δ^ Ctrl, and PLN-R14^Δ/Δ^ ASO25 mice at 5 (baseline), 7, and 21 weeks of age.

PLN-ASO or vehicle treatment started at 5 weeks of age, in accordance to a previous study,^8^ with four injections during the first week of treatment, followed by weekly injections of the indicated doses (*Figure 1A*). As expected, vehicle-treated R14^Δ/Δ^ mice reached the humane endpoint at 7 weeks of age, which is in agreement with previous studies.^5^ PLN-ASO treatment showed a dose dependent increase in survival of R14^Δ/Δ^ mice, with the 3 mg/kg group surviving until 9 weeks of age, while those receiving 7 mg/kg reached the humane endpoint and were terminated at 11 weeks of age (*Figure 1B*). Importantly, the higher ASO dose groups of 15 and 25 mg/kg did not reach the humane endpoint and remained viable until the end of the experiment, except for one mouse in the 15 mg/kg group. All WT mice remained viable until the end of the experiment (*Figure 1B*).

Investigation of cardiac function, using echocardiography, also revealed a clear dose–response effect in the R14^Δ/Δ^ mice (*Figure 1C–F*). From 5 weeks (start of treatment) to 7 weeks of age, a rapid decline in LVEF was observed in the vehicle-treated R14^Δ/Δ^ group (R14^Δ/Δ^ Ctrl), whereas the EF of WT mice remained >60% (*Figure 1C*). ASO treatment prevented heart function decline and with increasing doses, a more preserved EF was observed. Since it takes some time to reach sufficient depletion of PLN by ASO-mediated silencing, an immediate stabilizing effect on cardiac function was not expected. No additional echocardiograms were made of the R14^Δ/Δ^ mice receiving vehicle and 3 mg/kg of ASO, because these animals reached the humane endpoint within 2 weeks after the first follow-up measurement. As R14^Δ/Δ^ mice receiving 7 mg/kg of ASO became lethargic around 11 weeks of age, we performed an additional echo and this revealed a further decline in cardiac function compared to the previous analysis at 7 weeks of age (*Figure 1C*). Subsequently, these mice were terminated at this time point, because this group reached the predefined humane endpoint (group average EF < 20%). Importantly, the EF of the 15 and 25 mg/kg groups did not decline further after the first follow-up echocardiogram (7 weeks of age), indicating stabilization of cardiac function (*Figure 1C*). This was even more profound in the highest dose group, suggesting that at this dose even some improvement may occur. LV end-systolic and end-diastolic internal diameters were significantly increased in vehicle-treated R14^Δ/Δ^ mice as compared to WT mice, indicating ventricular dilatation, which was dose-dependently rescued by PLN-ASO administration (*Figure 1D* and *E*). All observed effects were specific to PLN-ASO administration, as R14^Δ/Δ^ mice injected with a scrambled ASO at the highest dose (25 mg/kg) showed the same rapid decline in cardiac function as untreated mice with an average EF of 13.0 ± 1.1%, and reached the humane endpoint by 7 weeks of age (see Supplementary material online, *Figure S1*). Together, these data demonstrate that PLN-ASO therapy exerts a dose-dependent effect on cardiac function and lifespan in PLN-R14^Δ/Δ^ mice.

### 3.2 PLN depletion effectively mitigates cardiac remodelling in a dose-dependent manner

**Figure 2:**
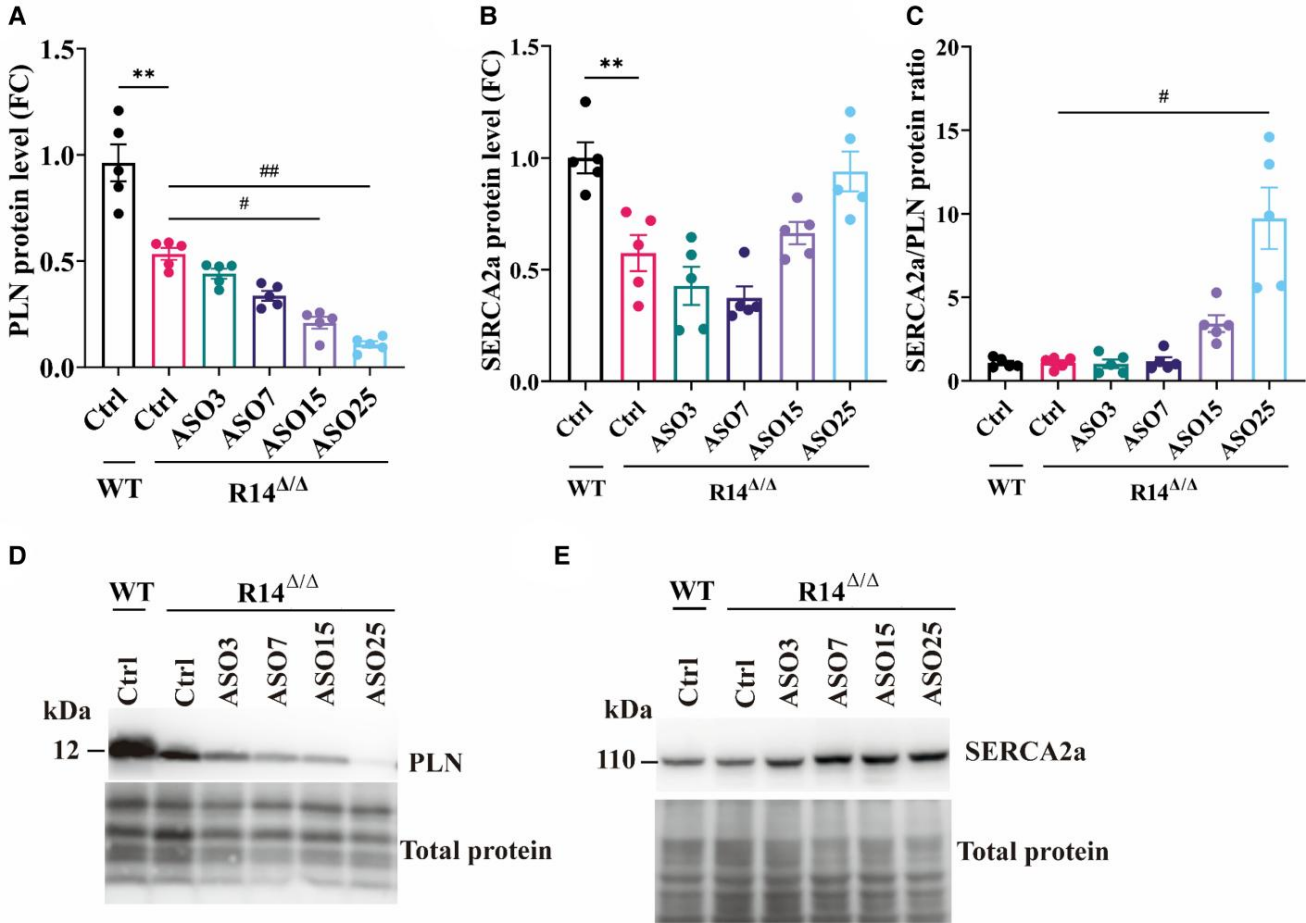
PLN-ASO administration effectively reduces the expression of PLN and increases the SERCA2a/PLN ratio in a dose-dependent manner. Protein quantification of (*A*) PLN and (*B*) SERCA2a and (*C*) their ratio (SERCA2a/PLN) in ventricular tissue of vehicle-treated WT (Ctrl) and vehicle-treated PLN-R14^Δ/Δ^ (Ctrl) mice and PLN-ASO-treated (3, 7, 15, or 25 mg/kg) PLN-R14^Δ/Δ^ mice (*n* = 5 per group). Ventricular tissue was collected at 21 weeks of age, except for those groups that reached the humane endpoint earlier (R14^Δ/Δ^: Ctrl at 7 weeks, ASO3 at 9 weeks and ASO7 at 11 weeks). Protein expression normalized to total protein levels, is shown as fold change (FC) compared to WT Ctrl. Representative (average based) western immunoblot images of (*D*) PLN (top), (*E*) SERCA2a (top), and their total protein staining (bottom). Cardiac tissue from mice was collected at various ages for protein isolation, as outlined in the study design. **P < 0.01 (Mann–Whitney test), ^#^*P* < 0.05, ^##^*P* < 0.01 (Kruskal–Wallis with Dunn's multiple comparisons test).

Next, to confirm effective depletion of PLN following PLN-ASO treatment, PLN expression was determined at both mRNA and protein level. Since R14^Δ/Δ^ mice receiving vehicle or 3 or 7 mg/kg PLN-ASO did not survive until the 21-week time point, it is important to note that these cardiac samples are from time points corresponding to their maximal lifespan (see *Figure 1B*). As shown, both gene (see Supplementary material online, *Figure S2A*) and protein (*Figure 2A*) expression levels were dose-dependently decreased in hearts of ASO-treated R14^Δ/Δ^ mice. We like to note that at 7 weeks of age, PLN levels are also lower in the R14^Δ/Δ^ vehicle group as compared to the WT group (*Figure 2A* and Supplementary material online, *Figure S2A*). This has been observed before and is at least partially related to downregulation of PLN in this animal model, as present in this group at 7 weeks.^19^ At the protein level this difference may also partly be explained by differences in antibody binding to wild-type PLN and PLN-R14del and therefore we have to be cautious with direct protein comparisons between WT and R14^Δ/Δ^ groups. Moreover, cardiomyocyte density was approximately 25% lower in the R14^Δ/Δ^ vehicle group compared to WT, consistent with previously reported cell death, which may contribute to the observed reduction in PLN levels (see Supplementary material online, *Figure S3*). However, a direct comparison of immunohistochemical staining for PLN and cardiac troponin revealed a pronounced decrease in PLN expression within cardiomyocytes (see Supplementary material online, *Figure S3*). While some bias in this quantification cannot be excluded, due to certain PLN clusters being out of focus, western blot analyses corrected for cardiac troponin I and α-actinin levels also confirmed reduced PLN protein levels in the R14^Δ/Δ^ vehicle group (see Supplementary material online, *Figure S4*). These findings corroborate earlier reports of cellular PLN downregulation in the context of heart failure.^8^ The dose-dependent efficacy of ASO-mediated PLN protein depletion, calculated as per cent reduction compared to vehicle-treated R14^Δ/Δ^ mice, is shown in Supplementary material online, *Table S4*. Besides PLN, also SERCA2a protein and gene (*Atp2a2*) expression levels were investigated (*Figure 2B*, Supplementary material online, *Figure S2B*). SERCA2a was reduced in the R14^Δ/Δ^ vehicle group and in the low dose ASO3 and ASO7 treatment groups, but was restored by the high dose ASO treatment. The observed reduction in SERCA2a is consistent with findings in both mice and human, where decreased SERCA2a expression is associated with heart failure.^20,21^ The restoration of SERCA2a expression in the high dose groups therefore aligns with improved cardiac function. Since a low SERCA2a/PLN ratio may contribute to heart failure,^20^ we also measured the ratio in this study. Although no significant decrease was observed in vehicle-treated R14^Δ/Δ^ mice, at high ASO doses SERCA2a/PLN ratios were increased (*Figure 2C*). Similar changes were also observed for SERCA2a gene expression and the ratio between gene expression levels of SERCA2a and PLN (see Supplementary material online, *Figure S2C*). Overall, these results confirm the expected dose-dependent reduction of cardiac PLN-R14del levels following PLN-ASO administration.

**Figure 3:**
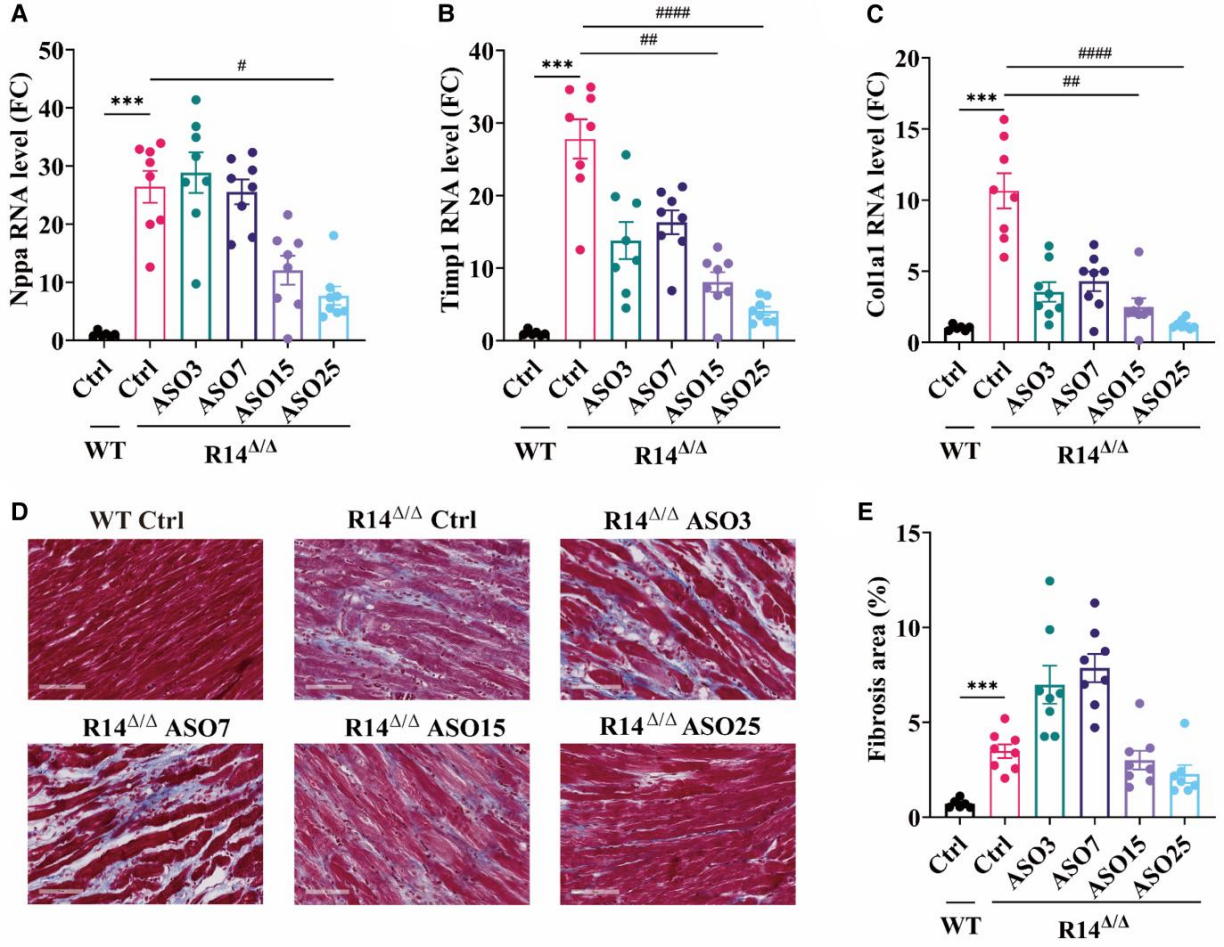
PLN depletion dose-dependently mitigates cardiac remodelling in hearts of PLN-R14^Δ/Δ^ mice. Ventricular gene expression of markers of cardiac remodelling. (*A*) atrial natriuretic peptide (*Nppa*), (*B*) Tissue Inhibitor of Metalloproteinases 1 (*Timp1*), and (*C*) collagen type I alpha 1 chain (*Col1a1*) of vehicle-treated WT (*n* = 6) and ASO-treated (3, 7, 15, or 25 mg/kg) PLN-R14^Δ/Δ^ mice (*n* = 8 per group), normalized to 36B4 (*Rplp0*) housekeeping gene levels and shown relative fold change to WT Ctrl. (*D*) Representative (average based) Masson's trichrome-stained cardiac tissue sections of vehicle-treated WT and ASO-treated (3, 7, 15, or 25 mg/kg) PLN-R14^Δ/Δ^ mice (scale bars, 70 μm). (*E*) Quantification of cardiac fibrosis in Masson's trichrome-stained cardiac tissue sections of vehicle-treated WT (*n* = 6) and ASO-treated (3, 7, 15, or 25 mg/kg) PLN-R14^Δ/Δ^ mice (*n* = 8 per group), displayed as percentage of total stained area. Ventricular tissue were collected at 21 weeks of age, except for those groups that reached the humane endpoint earlier (R14^Δ/Δ^: Ctrl at 7weeks, ASO3 at 9 weeks and ASO7 at 11 weeks). ***P < 0.001 (Mann–Whitney test), ^#^*P* < 0.05, ^##^*P* < 0.01, ^####^*P* < 0.0001 (Kruskal–Wallis with Dunn's multiple comparisons test).

Molecular changes related to cardiac remodelling were also investigated. Gene expression of the cardiac wall stress and hypertrophy biomarker atrial natriuretic peptide (encoded by the *Nppa* gene) was significantly elevated in R14^Δ/Δ^ vehicle group compared to WT (*Figure 3A*). Consistent with improved cardiac function in the 25 mg/kg PLN-ASO-treated R14^Δ/Δ^ groups, *Nppa* was significantly reduced as compared to the vehicle-treated R14^Δ/Δ^ group (*Figure 3A*). A similar dose–response effect was observed for fibrotic gene expression (*Timp1* and *Col1a1*), indicative of diminished fibrosis formation (*Figure 3B* and *C*). To corroborate this finding, histochemistry was performed, using Masson's trichrome staining to visualize myocardial fibrosis in ventricular sections (*Figure 3D*). As shown before,^5^ in vehicle-treated R14^Δ/Δ^ mice, cardiac fibrosis was significantly elevated as compared to WT mice (*Figure 3D* and *E*). Hearts from R14^Δ/Δ^ mice treated with 3 and 7 mg/kg of ASO showed more collagen deposition as compared to the vehicle-treated R14^Δ/Δ^ group. We believe that the increased lifespan of these low dose groups, as compared to the vehicle group, provides an increased time window for fibrosis development, ultimately resulting in higher levels of extracellular matrix. Most importantly, however, is that in fibrosis was not further elevated in the high dose groups. Thus, PLN-ASO therapy dose-dependently reduces natriuretic peptide expression and halts fibrosis formation in hearts of R14^Δ/Δ^ mice, corroborating our *in vivo* findings.

### 3.3 PLN silencing dose-dependently resolves detrimental SR clusters in PLN-R14^Δ/δ^ mice

**Figure 4:**
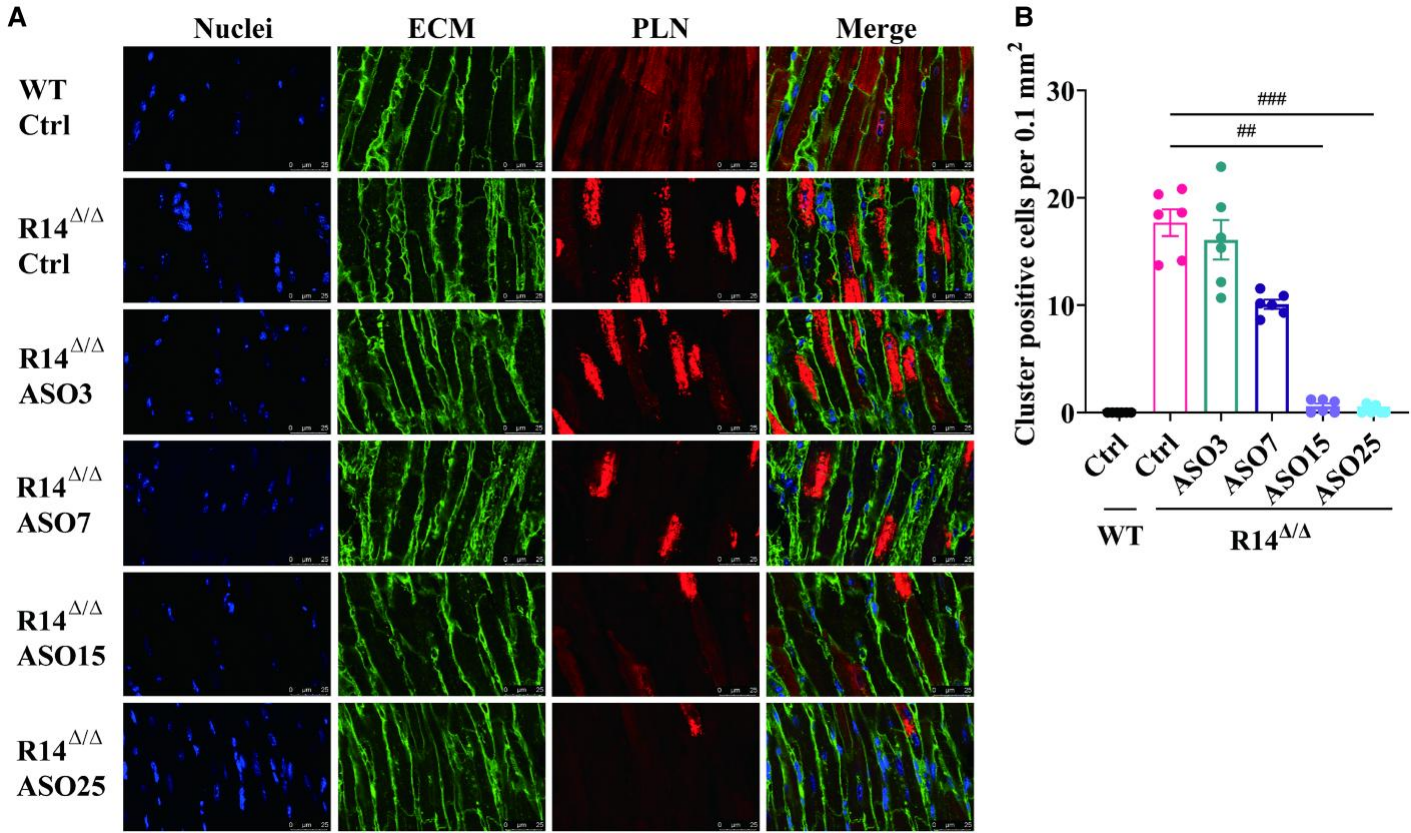
Silencing of PLN results in dose-dependent removal of abnormal intracardiomyocyte SR clusters in PLN-R14^Δ/Δ^ mice. (*A*) Representative (average based) immunofluorescence (IF) staining of PLN, together with wheat germ agglutinin (WGA) staining of extracellular matrix and 4′,6-diamidino-2-phenylindole (DAPI) staining of nuclei in ventricular tissue sections of vehicle-treated WT and ASO-treated (3, 7, 15, or 25 mg/kg) R14^Δ/Δ^ mice (scale bars, 25 μm). (*B*) PLN-cluster-positive cardiomyocytes per 0.1 mm^2^ of stained tissue sections (*n* = 6 per group). Statistical comparison between WT and R14^Δ/Δ^ mice receiving vehicle treatment was not performed as no SR clusters were found in WT hearts. ^##^*P* < 0.01, ^###^*P* < 0.001 (Kruskal–Wallis with Dunn's multiple comparisons test).

**Figure 5:**
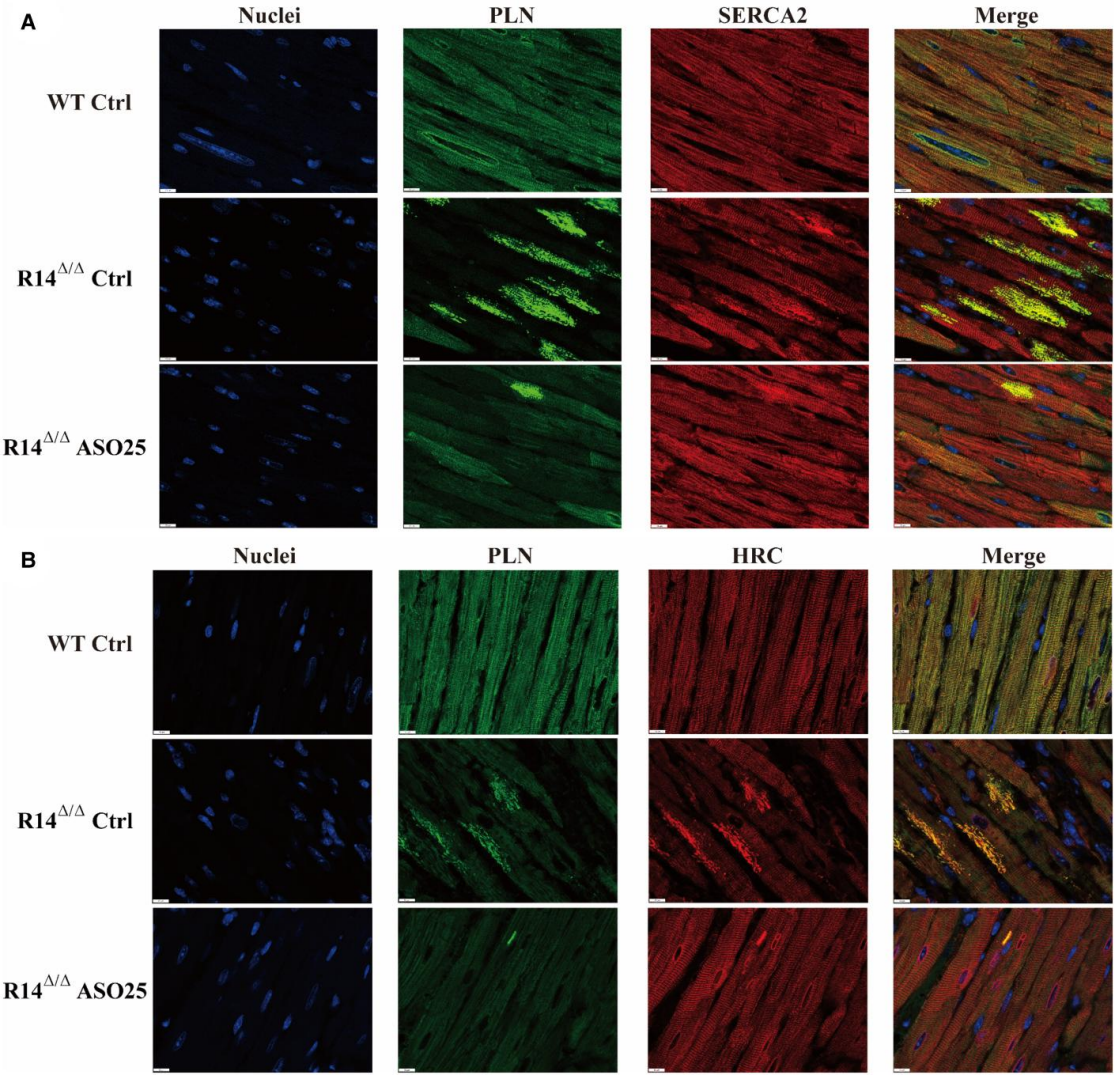
Treatment with PLN-ASO recovers SR organization in hearts of PLN-R14^Δ/Δ^ mice. Representative immunofluorescent double staining for PLN together with (*A*) SERCA2a (sarcoplasmic reticulum Ca^2+^ ATPase 2) or (*B*) HRC (histidine-rich calcium-binding protein) in ventricular tissue sections of WT Ctrl, R14^Δ/Δ^ Ctrl, and PLN-ASO (25 mg/kg) PLN-R14^Δ/Δ^ mice (scale bars, 10 μm). Nuclei are stained blue using 4′,6-diamidino-2-phenylindole (DAPI).

A key hallmark of PLN-R14del cardiomyopathy in humans and mice is the occurrence of detrimental cardiac SR clusters, as evidenced by strong perinuclear PLN staining in R14^Δ/Δ^ cardiomyocytes.^5,9^ In vehicle-treated R14^Δ/Δ^ mice, PLN clusters were highly prevalent with 17.7 ± 1.3 cluster-positive cells per 0.1mm,^2^ whereas such clusters were not observed in WT mice (*Figure 4A* and *B*). Importantly, these SR clusters were dose-dependently reduced after ASO treatment and were nearly absent at the highest doses. Absence of PLN-positive clusters suggests that the organization of the SR is restored. We previously demonstrated that HRC and SERCA2a colocalize with PLN in aberrant SR clusters.^9^ To confirm restructuring of reticulum membranes, we performed immunohistochemical double staining for SERCA2a/PLN and HRC/PLN in cardiomyocytes from both WT and R14^Δ/Δ^ mice treated with either vehicle or 25 mg/kg ASO, assessing protein colocalization. (*Figure 5A* and *B*). Confocal imaging validated colocalization of these proteins and showed enrichment in the SR clusters of R14^Δ/Δ^ mice. Importantly, ASO treatment normalized SERCA2a and HRC localization in the majority of R14^Δ/Δ^ cardiomyocytes, confirming that ASO treatment restored SR organization. As PLN is not completely silenced upon ASO administration, low amounts of PLN and small SR clusters were observed in some cardiomyocytes of ASO-treated R14^Δ/Δ^ mice. Together, these findings demonstrate that PLN-ASO therapy resulted in dose-dependent restoration of SR structure in hearts of PLN-R14^Δ/Δ^ mice.

### 3.4 PLN-ASO treatment normalized SR structure in PLN-R14^Δ/δ^ mice

**Figure 6:**
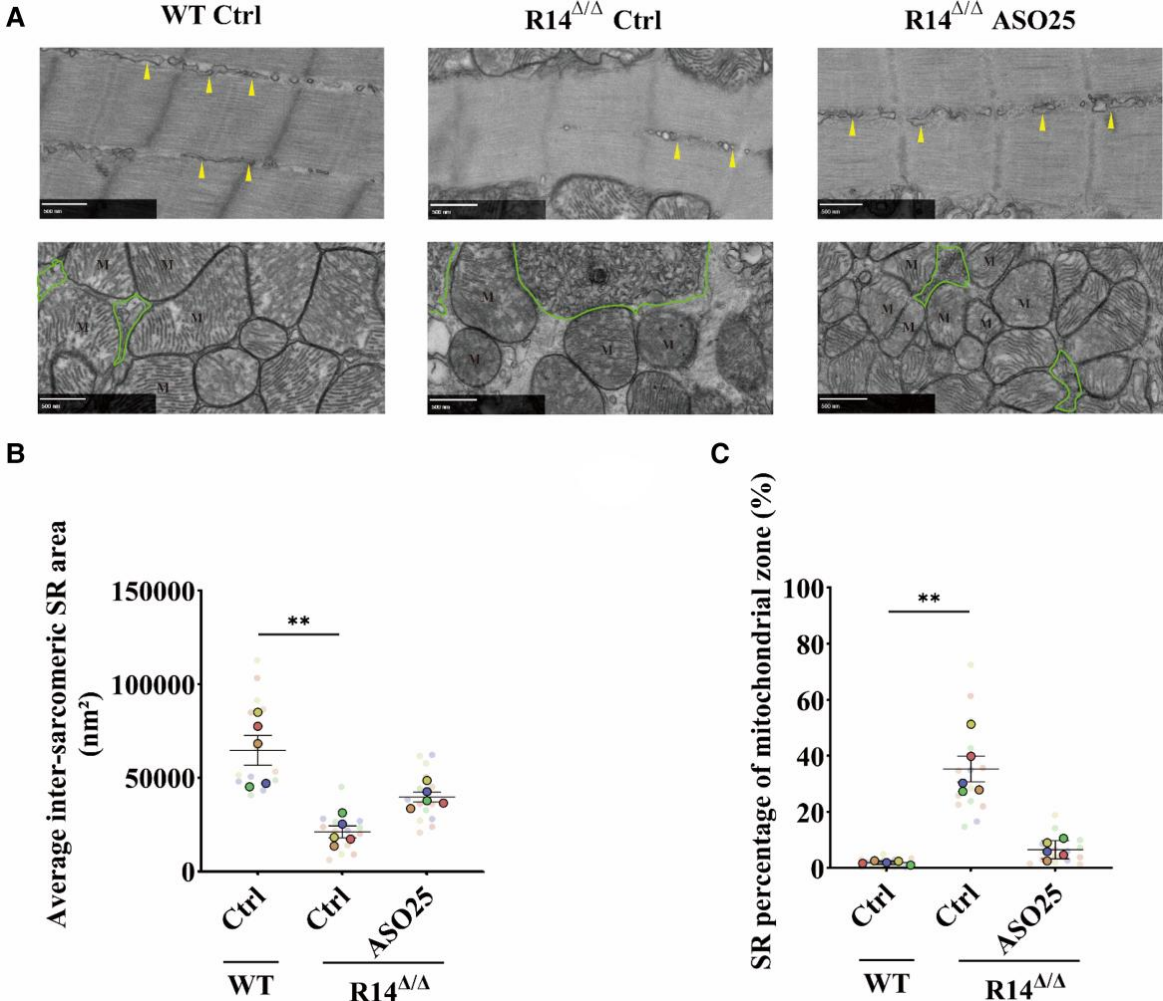
PLN-ASO treatment normalized SR structure in PLN-R14^Δ/Δ^ mice. (*A*) Representative electron microscopic images from cardiac ventricle of WT Ctrl, R14^Δ/Δ^ Ctrl, and PLN-ASO (25 mg/kg) PLN-R14^Δ/Δ^ mice (scale bars, 500 nm) showing a reduced SR area (arrow pointed) between adjacent sarcomeres and clustered SR (solid line circled) mislocalized around mitochondria (*M*). Quantification of SR area as the average SR area between adjacent sarcomeres (*B*) and a percentage within the mitochondrial zone (*C*). (*n* = 5 per group). *P < 0.05, ***P < 0.001, ****P < 0.0001 (one-way ANOVA test with Turkey's multiple comparisons test). In *Panels B* and *C*, the colours of the individual cell values (transparent dots) match the colours of the average values (solid dots) of the same mouse.

To visualize the sarcoplasmic reticulum (SR) structure in greater detail, electron microscopy (EM) was performed. Ventricular tissue from vehicle-treated wild-type (WT) mice and from both vehicle- and ASO25-treated R14^Δ/Δ^ mice (all terminated at 7 weeks of age) was analysed to assess SR restoration following ASO treatment (*Figure 6A–C*). In vehicle-treated R14^Δ/Δ^ mice, cardiomyocytes exhibited a markedly aberrant and SR distribution, in contrast to the well-organized SR network seen in WT mice. Notably, SR density between sarcomeres was significantly reduced (*Figure 6B*), while SR accumulation around mitochondria was increased and appeared disorganized (*Figure 6C*). Strikingly, irregular membrane-like structures were frequently observed near mitochondria, reflecting severe SR disarray. PLN-ASO25 treatment largely restored the normal SR architecture. Minimal distances between the SR and mitochondria, as well as the relative SR–T-tubule distances, were unchanged across groups (see Supplementary material online, *Figure S5*). Collectively, these findings indicate a pronounced remodelling of the longitudinal SR in R14^Δ/Δ^ mice, which is substantially reversed by ASO treatment.

### 3.5 The PLN-R14del pathogenic variant results in a partial loss of SERCA2a inhibitory function

**Figure 7:**
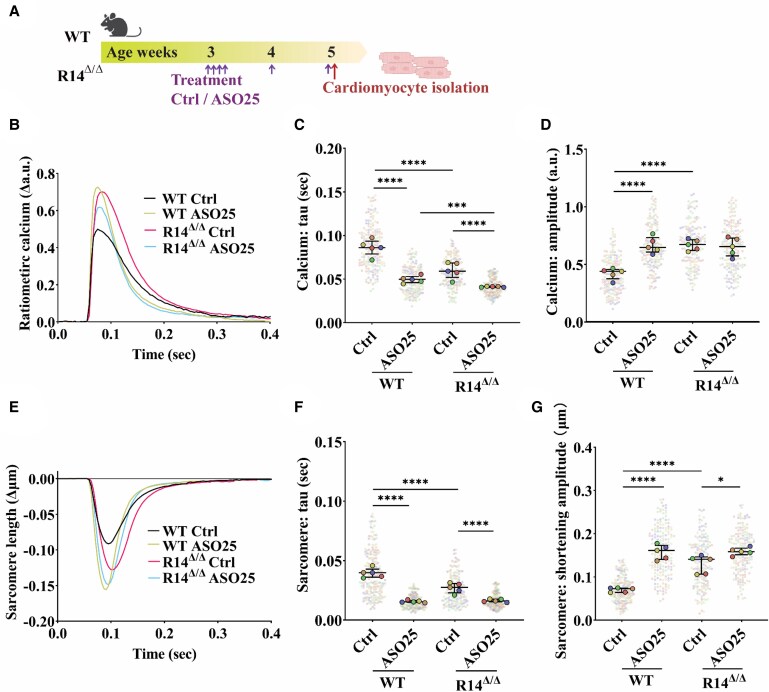
Isolated cardiomyocytes of PLN-R14^Δ/Δ^ mice demonstrate accelerated calcium decay and sarcomeric relaxation with limited effect of PLN depletion. (*A*) Schematic overview of the isolated cardiomyocyte study design. (*B*) Normalized ratiometric calcium transients under pacing conditions in cardiomyocytes isolated from WT and R14^Δ/Δ^ mice treated with vehicle or ASO (25 mg/kg; averaged transients of five mice per group). (*C*) Calcium decay time constant (tau) and (*D*) calcium amplitude (*n* = 5 per group). (*E*) Normalized sarcomere lengths under pacing conditions in cardiomyocytes isolated from WT and R14^Δ/Δ^ mice treated with vehicle or ASO (25 mg/kg; averaged transients of five mice per group). (*F*) Sarcomere decay time constant (tau) and (*G*) sarcomere shortening amplitude (*n* = 5 per group). *P < 0.05, ***P < 0.001, ****P < 0.0001 (hierarchical statistical test with Bonferroni correction).^17^ In *Panels C*, *D*, *F*, and *G*, the colours of the individual cell values (transparent dots) match the colours of the average values (solid dots) of the same mouse.

Since PLN regulates SERCA2a activity and the effect of PLN-R14del on SERCA2a has been debated, we investigated calcium cycling and sarcomere contraction in isolated cardiomyocytes of vehicle- and ASO-treated WT and R14^Δ/Δ^ mice. Due to the challenges of isolating cardiomyocytes in advanced disease, including the presence of extensive cardiac fibrosis and the frailty of diseased cardiomyocytes, cardiomyocyte isolation was performed at 5 weeks of age (an early disease stage). To reach sufficient PLN depletion, ASO treatment (with the same frequency as in the abovementioned experiment) was initiated at 3 weeks of age with the highest ASO dose (25 mg/kg) (*Figure 7A*). This 2-week treatment period resulted in strong reduction of PLN protein expression in cardiomyocytes isolated from WT and R14^Δ/Δ^ mice, as compared to the vehicle-treated mice of the same genotype (see Supplementary material online, *Figure S6*). For calcium measurements, cardiomyocytes were loaded with the ratiometric calcium fluorophore Fura-2, after which calcium transients and sarcomere shortening were measured simultaneously in single cells. As expected, silencing of PLN and subsequent relief of SERCA2a inhibition resulted in faster SR calcium uptake in ASO-treated WT cardiomyocytes as evidenced by significantly smaller tau (time constant of calcium transient decay) values and significantly greater calcium peak amplitudes (*Figure 7B–D*). In R14^Δ/Δ^ cardiomyocytes, tau was significantly lower and calcium transients were significantly higher than in WT cardiomyocytes, indicating a loss of SERCA2a inhibitory function in mice carrying the PLN-R14del pathogenic variant. Consistently, ASO-mediated depletion of PLN had a much smaller effect on calcium transient decay and did not alter calcium transient amplitudes in R14^Δ/Δ^ mice. The ASO effects on calcium cycling were also reflected in cardiomyocyte relaxation, resulting in significantly accelerated sarcomere relaxation (tau) and significantly increased shortening amplitude in WT cardiomyocytes (*Figure 7E–G*). R14^Δ/Δ^ cardiomyocytes demonstrated significantly smaller tau for sarcomere relaxation and sarcomeric shortening amplitude was significantly stronger as compared to WT cardiomyocytes. Consequently, the PLN silencing effects on contraction and relaxation were significant, though less pronounced in R14^Δ/Δ^ compared to WT cardiomyocytes. We also performed beta-adrenergic stimulation using isoproterenol, which had a profound effect on all indicated parameters in vehicle-treated WT cardiomyocytes (see Supplementary material online, *Figure S7*). The effect of isoproterenol is partially dependent on protein kinase A (PKA)-mediated phosphorylation of PLN, resulting in reduced SERCA2a inhibition and enhanced calcium cycling, contraction and relaxation.^22^ Not surprisingly, isoproterenol had only limited additive effect on these parameters in ASO-treated WT cardiomyocytes (see Supplementary material online, *Figure S7*). Similarly, isoproterenol had limited additive effects in R14^Δ/Δ^ cardiomyocytes with or without ASO treatment (see Supplementary material online, *Figure S7*), indicating that the R14del pathogenic variant of PLN is a partial loss-of-function mutation. Nevertheless, isoproterenol stimulation of ASO-treated WT and R14^Δ/Δ^ cardiomyocytes further increase calcium transient amplitude, showing that PLN depletion did not deplete cardiac reserve. Altogether, the current study shows that PLN-ASO therapy has limited effects on calcium handling in PLN-R14del cardiomyopathy.

## 4. Discussion

In this study, we showed a dose dependent treatment effect with PLN-ASO on cardiac remodelling and survival in a murine PLN-R14del cardiomyopathy model. Our results revealed that PLN-R14^Δ/Δ^ mice benefit already from ASO treatment at low doses (3 and 7 mg/kg) with a delay in cardiac functional decline and death. At higher doses (15 and 25 mg/kg), cardiac function was stabilized and lifespan was prolonged from 7 weeks to 21 weeks (the predefined endpoint of the experiment). Detrimental SR cluster formation, a hallmark of PLN-R14del cardiomyopathy,^9,23^ showed a corresponding dose-dependent reduction. Investigation of other SR marker proteins showed that PLN depletion restored SR structure in R14^Δ/Δ^ mice. Finally, we demonstrated that sarcomere relaxation and SR Ca^2+^ uptake were accelerated in isolated cardiomyocytes from WT mice after ASO treatment. In R14^Δ/Δ^ mice, however, these parameters were already enhanced and ASO treatment had limited additional effect. This indicates that the PLN-R14del mutation is a partial loss-of-function variant. Therefore, the beneficial ASO effects do not primarily relate to enhanced SERCA2a-mediated calcium transport. Instead, the PLN-R14del mutation appears to gain a pathogenic function, disrupting normal SR structure, which ASO treatment helps to restore.

Previously, we have demonstrated that a high dose of ASO (≥50 mg/kg) significantly improved survival in R14^Δ/Δ^ mice.^7,8^ While this demonstrated proof of concept, we had not yet explored the dose–response effects or the underlying mechanisms. One proposed mechanism is the relief of PLN-R14del-mediated superinhibition of SERCA2a following PLN depletion. Normally, phosphorylation of PLN by PKA alleviates SERCA2a inhibition, but PLN-R14del lacks the PKA consensus site and cannot be phosphorylated upon beta-adrenergic stimulation.^24^ This led to the suggestion that PLN-R14del might constitutively inhibit SERCA2a.^10^ However, while some studies support this idea, recent evidence suggests that PLN-R14del is a (partial) loss-of-function mutation with reduced or no inhibitory activity on SERCA2a.^12,25^ If the PLN-R14del variant results in loss of inhibitory function, further alleviation of SERCA2a inhibition by ASO-mediated depletion of PLN would have limited therapeutic benefit. To investigate this further, we examined calcium cycling and contraction in isolated cardiomyocytes from WT and R14^Δ/Δ^ mice, with and without ASO treatment, and in the presence and absence of beta-adrenergic stimulation. Consistent with recent studies,^9,12^ we found that R14^Δ/Δ^ cardiomyocytes exhibit enhanced SR calcium uptake compared to WT cardiomyocytes, indicating that PLN-R14del has reduced SERCA2a inhibitory activity. The effect of ASO treatment on calcium transient decay in R14^Δ/Δ^ cardiomyocytes was therefore less pronounced, supporting the notion that PLN-R14del is a partial loss-of-function mutation. Additional studies with beta-adrenergic stimulation (isoproterenol) showed positive inotropic effects in WT cardiomyocytes consistent with, amongst others, PKA-mediated PLN phosphorylation relieving SERCA2a inhibition.^9^ However, the response to isoproterenol stimulation was reduced in R14^Δ/Δ^ cardiomyocytes, because these cells showed enhanced SR calcium uptake at baseline, similar to findings in PLN knockout (KO) mouse cardiomyocytes.^26,27^ Enhanced calcium dynamics were reflected by improved sarcomere relaxation parameters in R14^Δ/Δ^ cardiomyocytes. Overall, our results provide evidence that PLN-R14del is a partial loss-of-function mutation.

As mentioned above, cardiomyocytes from PLN-KO mice exhibit accelerated SR calcium uptake,^26,27^ similar to what we observed in PLN-R14del cardiomyocytes. Additionally, the inotropic response to beta-adrenergic stimulation is either absent or nearly absent in both PLN-KO and PLN-R14del cardiomyocytes.^26,27^ However, while PLN-KO mice are viable and age normally up to 24 months,^28^ PLN-R14^Δ/Δ^ mice develop severe HF within 8 weeks after birth. This indicates that loss of SERCA2a inhibitory function of PLN-R14del cannot account for the rapid cardiac decline and shortened lifespan observed in these mice. In agreement with others,^9,11,12^ this suggests that PLN-R14del has acquired a novel pathogenic function. Abnormal perinuclear localization of PLN has been identified as a hallmark of PLN-R14del cardiomyopathy, and we have recently demonstrated that this abnormality is associated with structurally altered SR.^9^ Cardiomyocytes with these abnormal SR structures eventually undergo necrosis, leading to replacement fibrosis.^9^ Treatment with ASO resulted in a dose-dependent reduction of perinuclear PLN staining, with other SR markers also returning to normal localization post-treatment. Therefore, the induction of abnormal PLN-SR clustering represents a novel pathological gain-of-function of the PLN-R14del protein, which explains why reducing this protein through ASO therapy effectively halts disease progression.

In this study, we established, for the first time, a clear dose–response relationship between ASO treatment and PLN clustering and survival in PLN-R14^Δ/Δ^ mice. Even with a limited reduction of PLN, there was a delay in the decline of cardiac function and a modest extension of survival in the R14^Δ/Δ^ mouse model. Importantly, humans with the heterozygous PLN-E2stop (p.Glu2Ter) variant, showing a 50% reduction in PLN levels are healthy.^29^ A single homozygous individual from consanguineous parents did, however, developed DCM. However, the generalizability of this finding is unclear, as animal studies have shown normal cardiac function in the absence of PLN under both rest and stress conditions.^27,30^ Collectively, this highlights that partial PLN reduction through silencing can have therapeutic potential in humans. Our results demonstrate that reducing PLN-R14del is beneficial without full elimination, even in this aggressive R14^Δ/Δ^ model, underscoring the viability of partial PLN reduction as a treatment strategy.

We acknowledge several limitations of this study. First, the use of mice as a model organism to study PLN pathophysiology has inherent challenges. While PLN function is well conserved between humans and mice, there may be differences in the exact contribution of PLN in calcium homeostasis in each species. Additionally, we used homozygous PLN-R14del mice, whereas human patients are heterozygous for this variant. The decision to use homozygous PLN-R14del mice was driven by their accelerated disease progression and the demonstrated efficacy of ASO treatment in prior studies. Importantly, these homozygous mice also recapitulate most of the key pathological features observed in the human disease.^5^ Although, initially, it was suggested that PLN-R14del would localize to the plasma membrane in the absence of wild-type PLN,^31^ more recent studies have not confirmed this.^9,32^ This has recently been discussed and provides further support for the use of a homozygous mouse model for accelerated and synchronous disease development.^6^ Human PLN-R14del induced pluripotent stem cell (iPSC)-derived cardiomyocytes have been studied, including viral miRNA-mediated PLN silencing (∼50% reduction).^33^ However, in these *in vitro* models, it is challenging to recapitulate the key phenotypes observed in patients, including abnormal SR clustering; thus, therapeutic validation in such *in vitro* models still has limitations.^6^

In conclusion, this study demonstrates a dose-dependent protective effect of ASO treatment in PLN-R14del cardiomyopathy mice. This protective effect does primarily not result from improved calcium cycling, but is likely due to a reduction in abnormal SR structures caused by the altered pathogenic function of the PLN-R14del protein.

Translational perspective Human carriers of a p.Arg14del (R14del) variant of the phospholamban (PLN) gene face high risk of developing dilated and/or arrhythmogenic cardiomyopathy, progressing to severe heart failure. As patients are mostly non-responsive to standard care and no specific treatment for exists, heart transplantation is often a last option. PLN antisense therapy shows promise as the first precision medicine, targeting the root cause of the disease. Our data indicate that, at optimal dosing, PLN antisense therapy halts disease progression and extends lifespan in mice. While PLN-ASO enhances calcium cycling, its primary therapeutic effect in PLN-R14del cardiomyopathy is by reducing harmful PLN-SR clusters.

## Supplementary Material

cvaf156_Supplementary_Data

## Data Availability

All data that support the findings of this study are available from the corresponding author upon reasonable request.
