# Supplementary material for: In PLN-R14del mice, SR structure restoration, rather than calcium cycling, is the dominant effector of PLN-ASO treatment
Source: Cardiovasc Res. 2025 Sep 4;121(13):2042–54. doi: 10.1093/cvr/cvaf156 (PMC12560789; doi:10.1093/cvr/cvaf156)
Supplement: cvaf156_Supplementary_Data [file cvaf156_supplementary_data.zip › supplement revision20250607.docx]

**SUPPLEMENTARY DATA**

**In PLN-R14del mice, SR structure restoration, rather than calcium cycling, is the dominant effector of PLN-ASO treatment**

**Short title:** ASO treatment improves SR structure in PLN-R14del

Liu Sun^1^, Tim R. Eijgenraam^1^, Carl Amilon^2^, David Janzén^2,3^, Kenny M. Hansson^4^, Dieter A. Kubli^5^, Daniela Später^4^, Adam E. Mullick^5^, Peter van der Meer^1^, Vivian Oliveira Nunes Teixeira^1^, Herman H.W. Silljé^1^*

^1^Department of Cardiology, University Medical Center Groningen, University of Groningen, Groningen, The Netherlands

^2^Drug Metabolism and Pharmacokinetics, Research and Early Development, Cardiovascular, Renal and Metabolism, BioPharmaceuticals R&D, AstraZeneca, Gothenburg, Sweden

^3^Current affiliation: Clinical Pharmacology & Quantitative Pharmacology CVRM, Clinical Pharmacology and Safety Sciences, AstraZeneca R&D, Gothenburg, Sweden

^4^Bioscience Cardiovascular, Research and Early Development, Cardiovascular, Renal and Metabolism (CVRM), BioPharmaceuticals R&D, AstraZeneca, Gothenburg, Sweden

^5^Ionis Pharmaceuticals, Carlsbad, CA, USA

***Corresponding author**

Herman H. W. Silljé, PhD

Antonius Deusinglaan 1, 9713 AV, Groningen, The Netherlands

Phone: +31 (0)50 361 53 39

Fax: +31 (0)50 361 55 25

Email: h.h.w.sillje@umcg.nl **Supplementary Figures**


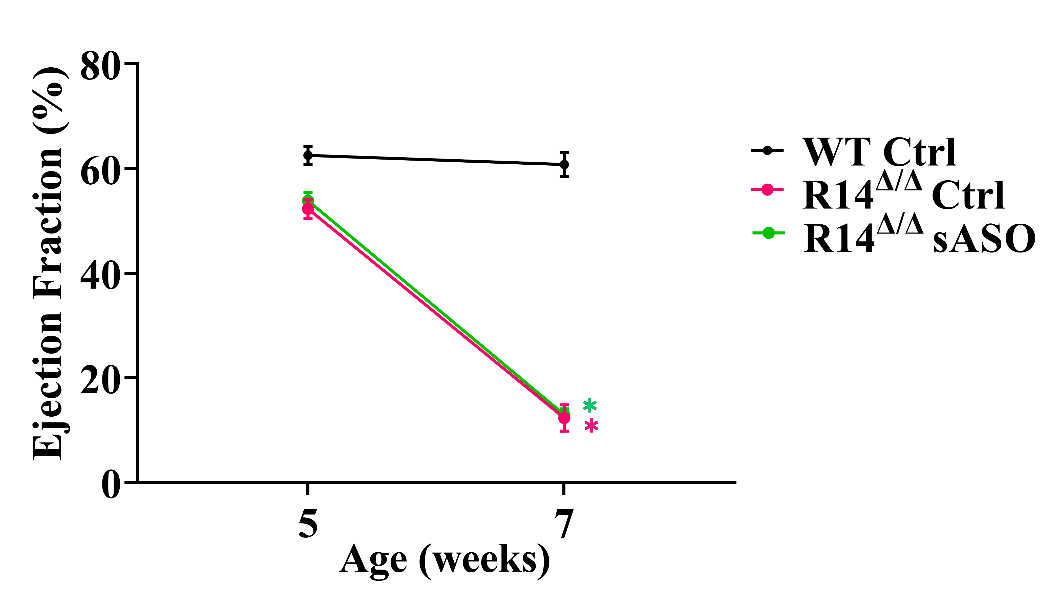


**Supplementary Figure S1. Scrambled** **ASO administration does not alter cardiac function of PLN-R14^∆/∆^ mice.**

Ejection fraction of WT Ctrl (n=6) and R14^∆/∆^ Ctrl or scrambled ASO (sASO) (n=8 per group) at 5 and 7 weeks of age. *p<0.05 vs. WT mice of the same age (Kruskal-Wallis with Dunn's multiple comparisons test).


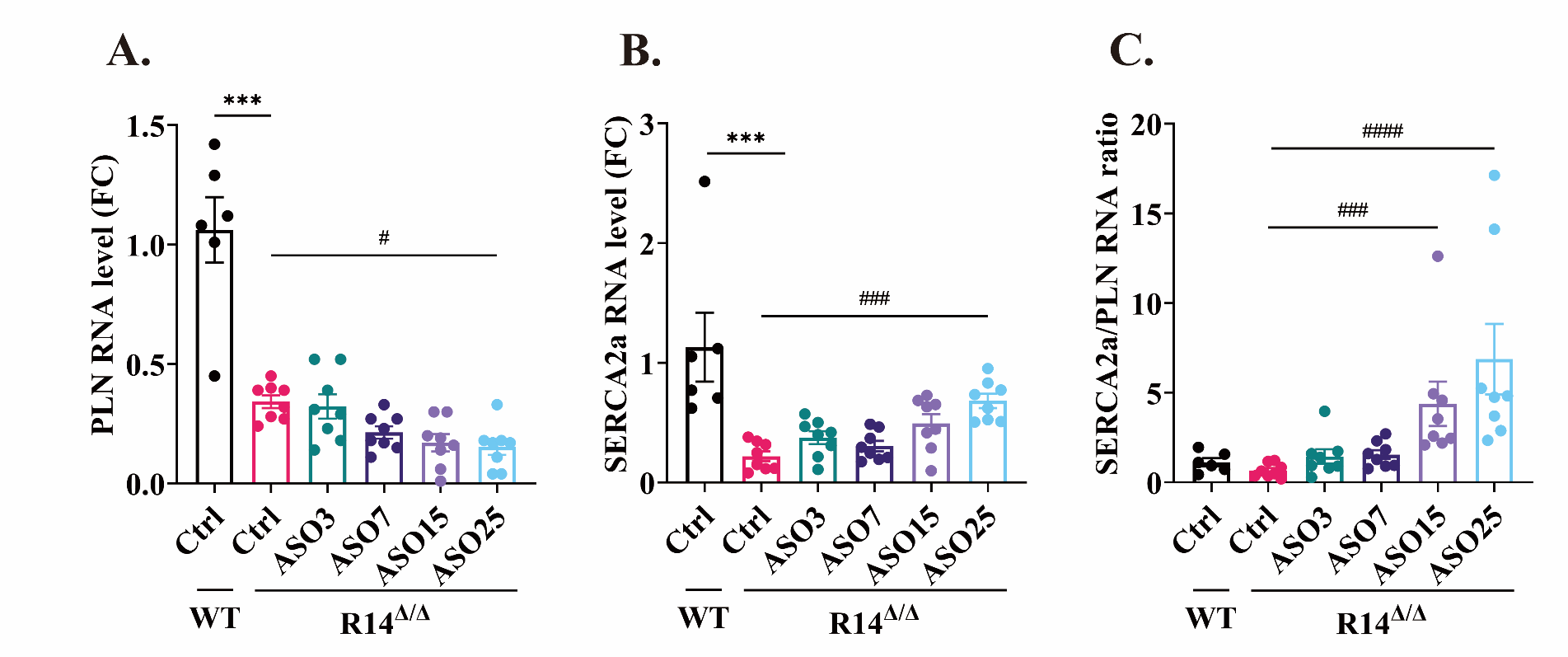


**Supplementary Figure S2. PLN-ASO administration effectively reduces the RNA level of PLN and increases the SERCA2a/PLN ratio in a dose-dependent manner.**

Relative ventricular mRNA levels of **(A)** PLN (*Pln*) and **(B)** SERCA2a (*Atp2a2*), normalized to housekeeping gene *Rplp0* (36B4), and **(C)** SERCA2a/PLN ratio of vehicle-treated WT (n=6), PLN-R14^∆/∆^ mice (n=8) and PLN-ASO-treated (3, 7, 15 or 25 mg/kg) R14^∆/∆^ mice (n=8), depicted as fold changes (FC) compared to WT Ctrl. Ventricular tissue was harvested at the predetermined termination time points, either upon reaching the humane endpoint or at 21 weeks of age. ***p<0.001 (Mann-Whitney test), ^#^p<0.05, ^###^p<0.001, ^####^p<0.0001 (Kruskal-Wallis with Dunn’s multiple comparison test).

**Supplementary Figure S3. Cardiomyocyte cell density and PLN expression decreased in R14^∆/∆^ vehicle group**

Cardiomyocyte cell density and PLN expression in WT and R14^∆/∆^ vehicle groups determined by immunofluorescence microscopy (A). Cardiomyocyte density was determined by counting the number of cardiac Troponin I stained cells in a transverse cut section and corrected by the area. n=6 per group. (B) The amount of PLN fluorescence and cardiac Troponin I fluorescence were determined in cardiomyocytes in a longitudinal cut section. The ratio between PLN and cardiac Troponin I are shown for the WT and R14^∆/∆^ groups (n=6). *p<0.05, **p<0.01 (Mann-Whitney test).

**
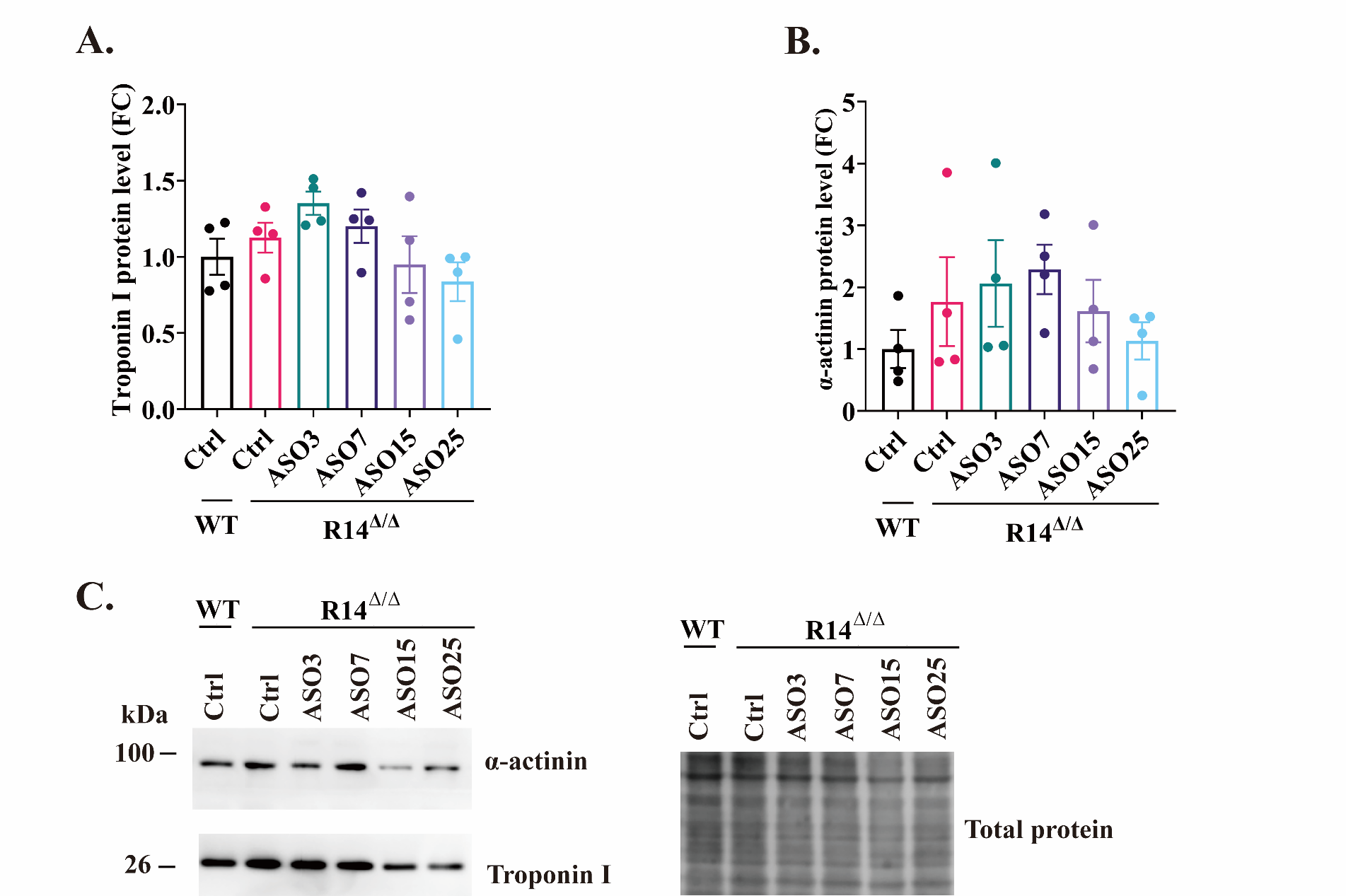
**

**Supplementary Figure S4. Cardiac expression of troponin I and α-actinin is not affected by heart failure.**

Protein quantification of **(A)** cardiac troponin I and **(B)** α-actinin in ventricular tissue of vehicle-treated WT (Ctrl) and R14^∆/∆^ (Ctrl) mice and PLN-ASO-treated (3, 7, 15 or 25 mg/kg) R14^∆/∆^ mice (n=4 per group). Protein expression was normalized to total protein levels and shown as fold change (FC) compared to WT Ctrl. **(C)** Representative (average based) Western immunoblot images of α-actinin (left top), troponin I (left bottom) and total protein staining (right). The PVDF membrane was cut into sections based on the expected molecular weights of the target proteins, and each section was incubated with specific antibody accordingly.

**
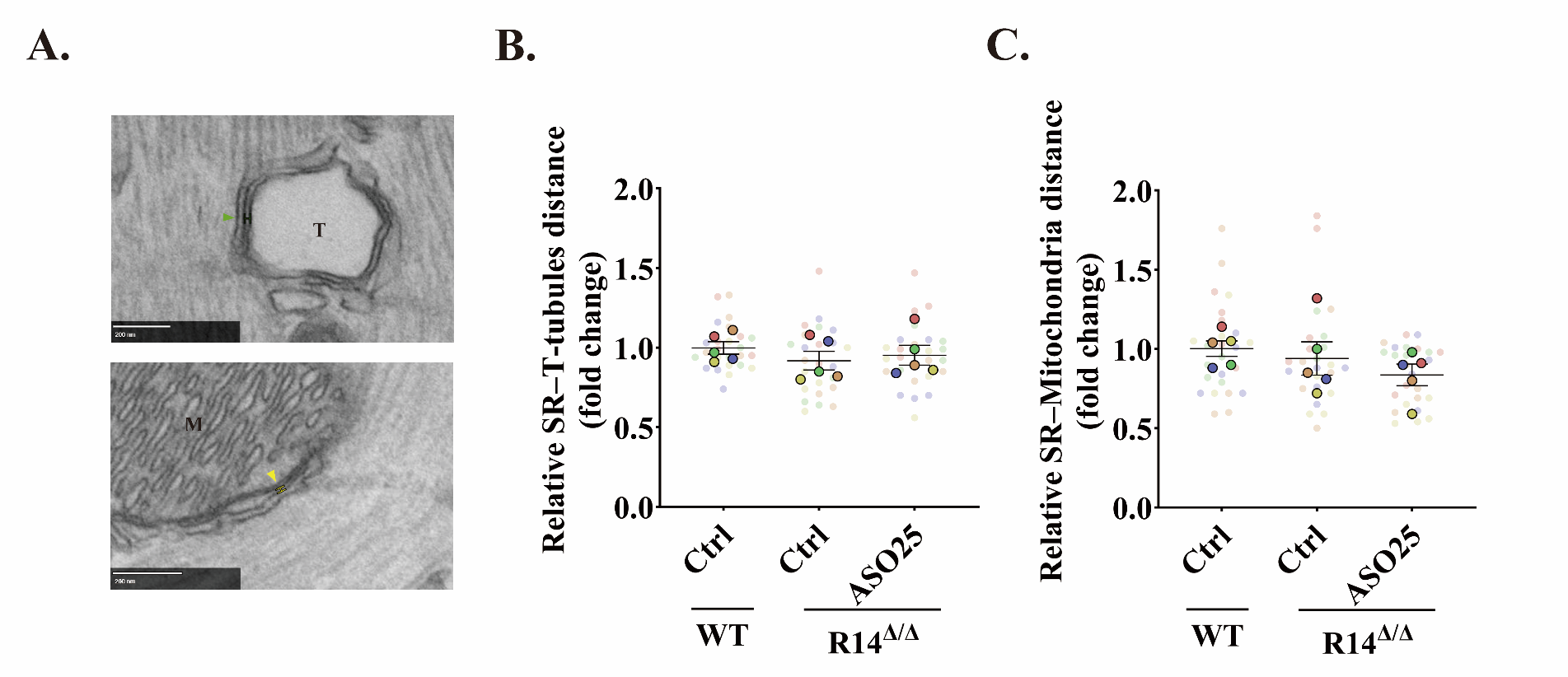
**

**Supplementary Figure S5. PLN- R14^∆/∆^ mice exhibit preserved spatial association between the SR, T-tubules, and mitochondria.**

**(A)** Representative electron micrographs showing the measured distances between the SR and T-tubules (top, green bar) and between the SR and mitochondria (bottom, yellow bar) (scale bar 200nm). **(B-C)** Quantification of the relative distance between SR–T-tubules **(B)** and SR–mitochondria **(C)**. (n = 5 per group). In panels B, C the colors of the individual cell values (transparent dots) match the colors of the average values (solid dots) of the same mouse.


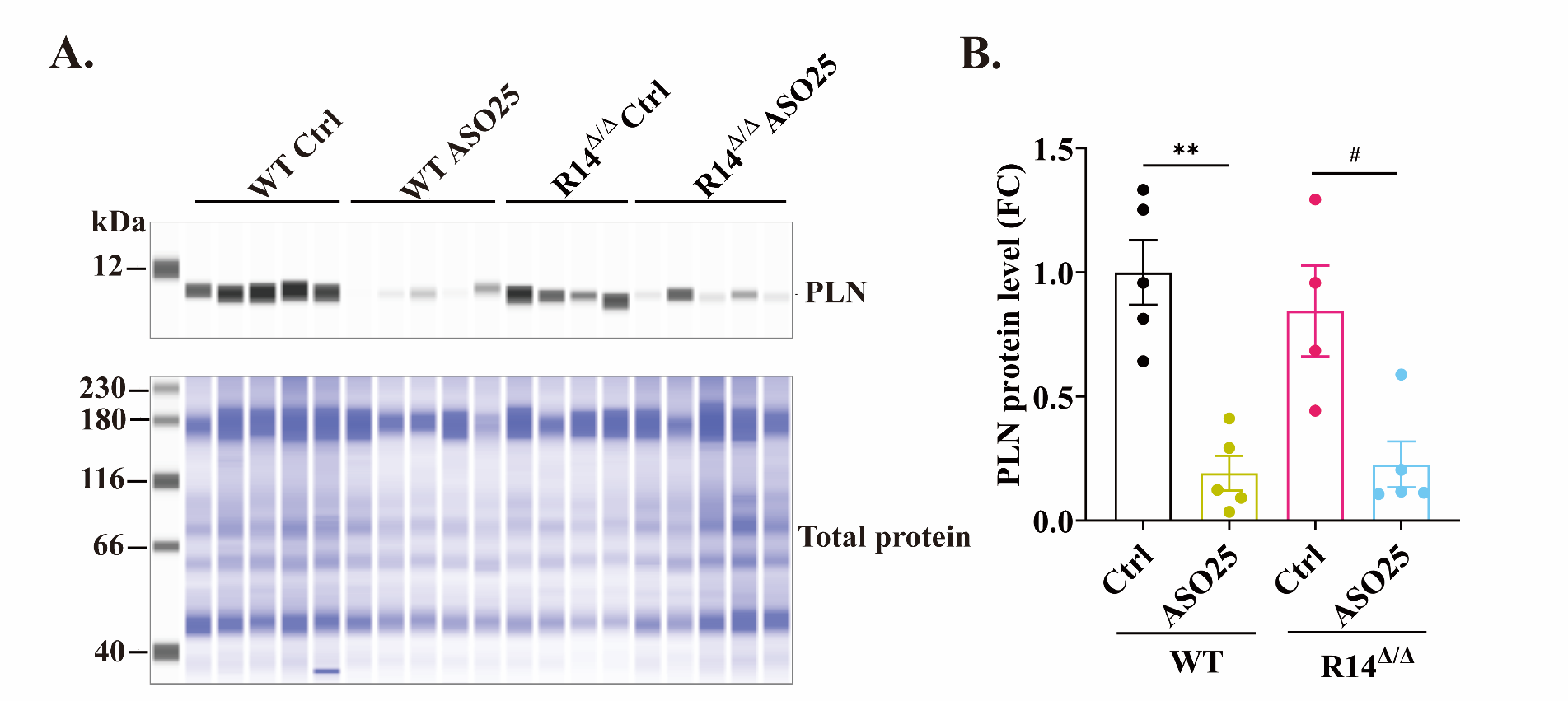


**Supplementary Figure S6. Efficient knockdown of PLN protein in isolated cardiomyocytes following PLN-ASO administration.**

Jess Western immunoblot images of **(A)** PLN (top), and its total protein (bottom) **(B)** PLN protein levels in cardiomyocytes isolated from WT and R14^Δ/Δ^ mice treated with vehicle or 25 mg/kg PLN-ASO, normalized to total protein levels and shown as fold change (FC) compared to vehicle-treated WT cardiomyocytes (n=5 per group, except n=4 for vehicle-treated R14^Δ/Δ^ cardiomyocytes). **p<0.01, #p<0.05 (Mann-Whitney test).

**
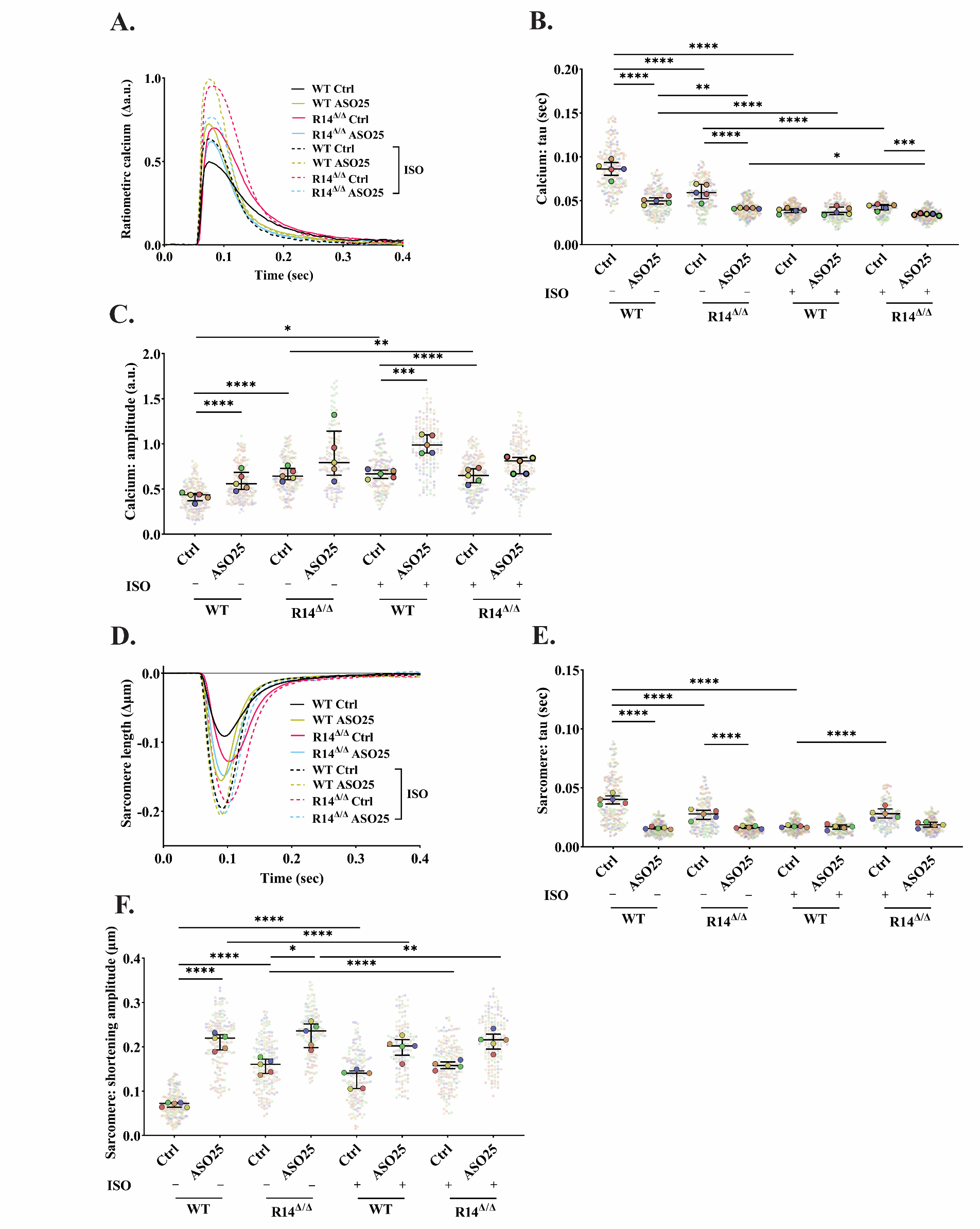
**

**Supplementary Figure S7. Isolated cardiomyocytes from PLN-R14^Δ/Δ^ mice exhibit enhanced contractile and calcium dynamics, with limited response to isoproterenol stimulation.**

**(A)** Normalized ratiometric calcium transients under pacing conditions in cardiomyocytes isolated from WT and R14^Δ/Δ^ mice treated with vehicle or PLN-ASO (25 mg/kg; n=5 per group), before (solid lines) and after addition (dashed lines) of isoproterenol (ISO). **(B)** Calcium decay time constant (tau) and **(C)** calcium amplitude (n=5 per group). **(D)** Normalized sarcomere lengths under pacing conditions in cardiomyocytes isolated from WT and R14^Δ/Δ^ mice treated with vehicle or PLN-ASO (25 mg/kg; n=5 per group), before (solid lines) and after addition (dashed lines) of isoproterenol (ISO). **(E)** Sarcomere decay time constant (tau) and **(F)** sarcomere shortening amplitude (n=5 per group). *p<0.05, **p<0.01, ***p<0.001, ****p<0.0001 (hierarchical statistical test with Bonferroni correction).^17^ In panels B, C, E and F, the colors of the individual cell values (transparent dots) match the colors of the average values (solid dots) of the same mouse.

**Supplementary Tables**

**Supplementary Table S1. Antibodies used for histological staining.**

| **Primary antibody** | **Host** | **Cat. No.** | **Supplier** | **Dilution** |
| --- | --- | --- | --- | --- |
| Anti-PLN | Rabbit | ab219626 | Abcam | 1:1,000 |
| Anti-PLN | Mouse | MA3-922 | Thermo Fisher Scientific | 1:200 |
| Anti-SERCA2 | Mouse | MA3-919 | Thermo Fisher Scientific | 1:100 |
| Anti-HRC | Rabbit | HPA004833 | Sigma | 1:100 |

| **Secondary antibody** | **Antigen** | **Label** | **Cat. No.** | **Supplier** | **Dilution** |
| --- | --- | --- | --- | --- | --- |
| Donkey anti-rabbit | Rabbit | Alexa488 | A21206 | Thermo Fisher Scientific | 1:100 |
| Donkey anti-mouse | Mouse | Alexa555 | A31570 | Thermo Fisher Scientific | 1:100 |
| Donkey anti-mouse | Mouse | Alexa488 | A21202 | Thermo Fisher Scientific | 1:100 |
| Donkey anti-rabbit | Rabbit | Alexa555 | A31572 | Thermo Fisher Scientific | 1:100 |

**Supplementary Table S2. Primer sequences used for qPCR.**

| **Gene** | **Protein** |  | **Sequence (5’-3’)** |
| --- | --- | --- | --- |
| *Pln* | Phospholamban | Forward  Reverse | GACGATCACCGAAGCCAAG  CGAGCGAGTGAGGTATTGC |
| *Atp2a2* | Sarcoplasmic/endoplasmic reticulum Ca^2+^-ATPase 2 | Forward  Reverse | TCAGTATGACGGGCTTGTAG  CGGTAGCTTCTCCAACTTTC |
| *Nppa* | Atrial Natriuretic Peptide | Forward  Reverse | GCTTCCAGGCCATATTGGAG  GGTGGTCTAGCAGGTTCTTG |
| *Timp1* | Tissue Inhibitor of Metalloproteinases-1 | Forward  Reverse | CAACGAGACCACCTTATACC  CATATCCACAGAGGCTTTCC |
| *Col1a1* | Collagen type 1 alpha 1 chain | Forward  Reverse | AGAGCATGACCGATGGATTC  CGCTGTTCTTGCAGTGATAG |
| *Rplp0* | Ribosomal protein, large P0 (36B4) | Forward  Reverse | AACGGGTACAAACGAGTC  AGATGGATCAGCCAAGAAG |

**Supplementary Table S3. Antibodies used for Western blot.**

| **Primary antibody** | **Host** | **Cat. No.** | **Supplier** | **Dilution** | **Molecular weight (kDa)** |
| --- | --- | --- | --- | --- | --- |
| Anti-PLN | Rabbit | 14562S | Bioke | 1:1,000 | 12 |
| Anti-SERCA2 | Mouse | MA3-919 | Thermo Fisher Scientific | 1:1,000 | 110 |
| Anti-α-actinin | Mouse | A7811 | Sigma | 1:1,000 | 100 |
| Anti-Troponin | Rabbit | Ab47003 | Abcam | 1:2,000 | 26 |

| **Secondary antibody** | **Antigen** | **Label** | **Cat. No** | **Supplier** | **Dilution** |
| --- | --- | --- | --- | --- | --- |
| Rabbit anti-mouse | Mouse | HRP | P0260 | Dako | 1:2,000 |
| Goat anti-rabbit | Rabbit | HRP | P0448 | Dako | 1:2,000 |

**Supplementary Table S4. ASO-mediated PLN protein depletion.**

|  | **Mean of PLN protein depletion compared to PLN-R14^∆/∆^ Ctrl ± SEM** | **p value** |
| --- | --- | --- |
| PLN-R14^∆/∆^ Ctrl | 0.00 ± 5.37 |  |
| PLN-R14^∆/∆^ ASO3 | 17.59 ± 4.45 | ns |
| PLN-R14^∆/∆^ ASO7 | 37.09 ± 4.32 | ns |
| PLN-R14^∆/∆^ ASO15 | 60.79 ± 5.19 | 0.0127 |
| PLN-R14^∆/∆^ ASO25 | 79.88 ± 2.85 | 0.0005 |
