# Supplementary figures and images for: In PLN-R14del mice, SR structure restoration, rather than calcium cycling, is the dominant effector of PLN-ASO treatment
Source: Cardiovasc Res. 2025 Sep 4;121(13):2042–54. doi: 10.1093/cvr/cvaf156 (PMC12560789; doi:10.1093/cvr/cvaf156)

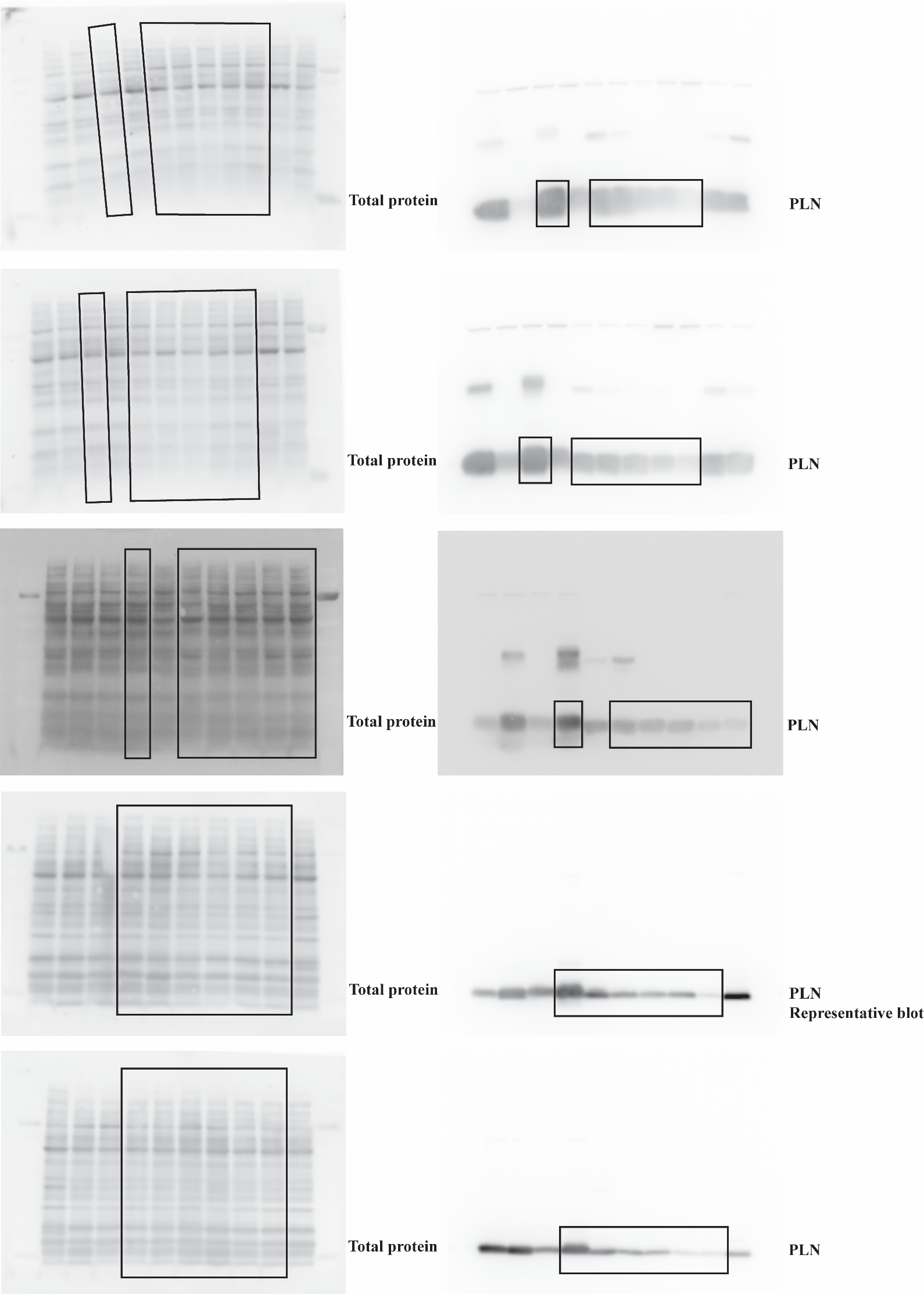

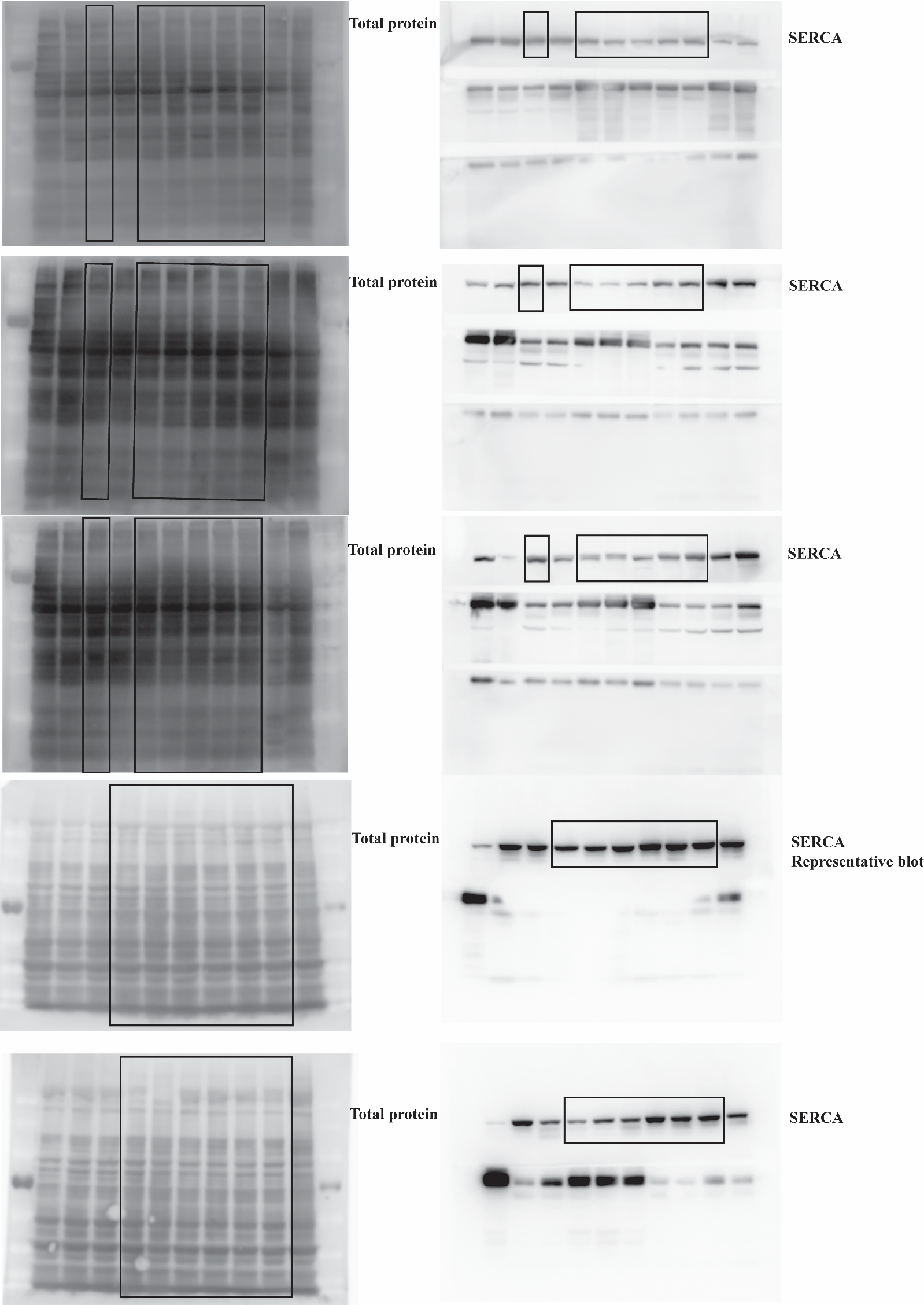

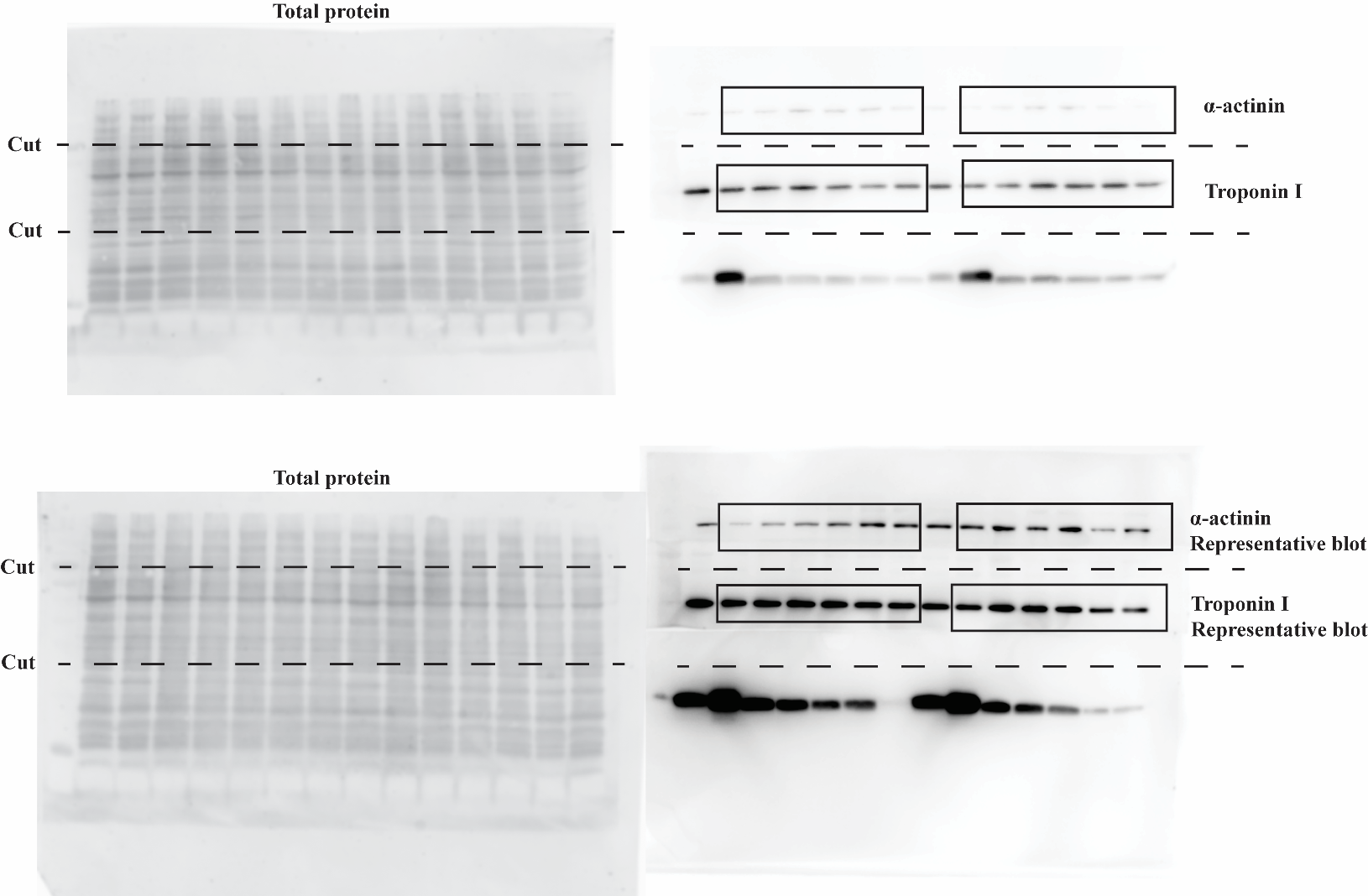

Supplement: cvaf156_Supplementary_Data [file cvaf156_supplementary_data.zip › supplement uncropped WB.docx]
